# Comprehensive nutritional compositions of traditional dishes from five Saudi regions using Elizabeth Stewart Hands and Associates Food Processor software

**DOI:** 10.3389/fnut.2025.1658078

**Published:** 2025-11-10

**Authors:** Reem Basaqr, El-Sayed Bakr, Yasmine Ibrahim, Yassmin Bujan, Kholoud Alqasimi, Abeer Aljaadi

**Affiliations:** 1 Department of Clinical Nutrition, College of Applied Medical Sciences, King Saud Bin Abdulaziz University for Health Sciences (KSAU-hs), Jeddah, Saudi Arabia; 2 King Abdullah International Medical Research Center, Ministry of the National Guard Health Affairs, Jeddah, Saudi Arabia; 3 Department of Nutrition and Food Sciences, Menofia University, Shebein El-kom, Egypt; 4 Department of Clinical Nutrition, Faculty of Applied Medical Sciences, Umm Al-Qura University, Makkah, Saudi Arabia; 5 Pathological Sciences Department-MBBS Program, Fakeeh College for Medical Sciences, Jeddah, Saudi Arabia; 6 Department of Medical Pharmacology, Faculty of Medicine, Minia University, El-Minia, Egypt; 7 Clinical Nutrition Department, Al-Salama Hospital, Jeddah, Saudi Arabia; 8 Clinical Nutrition Department, King Salman Specialized Hospital, Taif, Saudi Arabia

**Keywords:** nutrient, traditional Saudi dishes, ESHA, nutritional profile, cross-sectional study

## Abstract

**Introduction:**

Comprehensive nutritional information on the recipes of traditional Saudi dishes, an integral part of the country’s culinary heritage, is lacking. This study analyzed the nutritional composition of 25 commonly consumed traditional dishes from five Saudi Arabian regions using the Elizabeth Stewart Hands and Associates (ESHA) Food Processor Nutrition Analysis software.

**Methods:**

A cross-sectional study was conducted online using convenience sampling to identify commonly consumed traditional Saudi dishes. Overall, 360 individuals responded to the survey. Of these, follow-up phone interviews were conducted with 61 household recipe providers who had prepared the selected dishes at least five times in the past year and provided complete recipe data. Ingredients were weighed or converted to grams using food amount booklets. Nutrient values of the traditional dish recipes were estimated using the average of multiple recipes and software estimates per dish (a total of 75 recipes, three recipes for each cuisine), accounting for variations in ingredient amounts and preparation methods. The nutrient data are presented per 100 g and portion size.

**Results:**

The nutritional compositions of the selected dish recipes varied. The moisture, protein, fat, fiber, and carbohydrate contents were 5.7–80.4%, 3.4–13.0%, 2.0–13.3%, 0.26–5.8%, and 5.9–50.1%, respectively. The highest energy content per 100 g was found in Areekah (306.9 kcal) and the lowest was found in Margoug (89.2 kcal).

**Conclusion:**

Recipe-based ESHA estimation provided valuable insights into the nutritional value of traditional Saudi dish recipes, serving as a resource for dietary planning, public health initiatives, and future research. The nutritional profiles generated in this study may contribute to the development of accurate and culturally-appropriate dietary recommendations and exchange lists for Saudi Arabia.

## 1 Introduction

Saudi Arabian cuisine consists of diverse dishes that reflect regional traditions and variations (1, 2) contributing to a strong sense of identity and belonging. Dietary patterns in Saudi Arabia differ by sex, age, and geographic location (1, 3, 4), yet these habitual patterns are undergoing dramatic changes (5). Understanding the nutritional composition of foods is crucial for identifying potentially harmful ingredients, such as saturated fats, sodium, and added sugars, that negatively impact health. While traditional Saudi dishes are generally nutritious, many contain high levels of carbohydrates and saturated fat.

The nutrient content of foods widely consumed across different regions is influenced by multiple factors, including soil quality, climate, agricultural practices, food processing, and nutrient analysis methods. Thus, each country must maintain its own food composition database to ensure accuracy and relevance (6). Al-Walaan et al. generated proximate composition data for 27 traditional Kuwaiti/Middle Eastern dishes based on standardized recipes, yielding outputs suitable for inclusion in global food composition databases (7). Similarly, A Lebanese study analyzed macronutrients in 30 Lebanese traditional dishes using chemical procedures for analysis and translated them into meal-planning exchange lists (with Lebanese household measures) via the Wheeler method. Marked variability in macronutrients and fiber was observed (e.g., protein up to 29.7 g/100 g; fat 0.5–22.4 g/100 g), and exchange equivalents were established to support clinical dietetics and medical nutrition therapy (8). Although several countries have recently developed national food composition databases that are publicly accessible, few studies have analyzed Saudi dishes, and many of those available are outdated (9–11). For example, Al-Faris (12) reported the composition of 25 traditional Saudi dishes in 2017 but research covering different regions remains limited. Their study recommended further exploration of the nutritional contents of additional widely-consumed foods in Saudi Arabia.

Advanced nutritional analysis software, food composition tables, and laboratory-based assessments provide precise data on macronutrients and micronutrients. Access to a comprehensive food analysis database can be especially valuable when dining out, enabling individuals to estimate the nutritional composition of menu items and make informed choices that align with their health goals. For individuals with diabetes, establishing accurate nutritional information is particularly important for carbohydrate counting and long-term glycemic control.

The lack of updated data hinders individuals from making informed dietary decisions and limits the ability of healthcare practitioners to deliver accurate nutritional guidance. Developing clear, up-to-date resources is therefore essential to help individuals monitor their daily food intake (13). Given that most of the available literature on Saudi dishes is outdated, there is an urgent need for a reliable, updated food composition database that provides accurate information in a format that is accessible to clinicians and patients (2).

Accordingly, this study aimed to assess the nutritional profile of 25 traditional dish recipes commonly consumed across the five regions of Saudi Arabia. Using the Elizabeth Stewart Hands and Associates (ESHA) Food Processor Nutrition Analysis software, version 11.13x (Genesis R&D Food, released February 2023; ESHA Research Inc., Salem, OR, USA), we analyzed the approximate composition and nutritional content per 100 g of each dish. Our findings may support dietary planning, public health initiatives, and culinary research while promoting Saudi Arabia’s rich culinary heritage, ultimately providing accurate estimates of population-level dietary intake based on cultural preferences.

## 2 Materials and methods

### 2.1 Study design

We conducted a cross-sectional study using convenience sampling to select participants from the five Saudi regions from whom we collected cuisine-related data over a 2-month period. The study was approved by the Institutional Review Board of Fakeeh College for Medical Sciences (Reference No: 686/IRB/2024; Dated: May 26, 2024).

### 2.2 Participants and sampling

At the beginning of the study (Stage 1), an online survey was distributed through social media platforms, targeting Saudi residents across the five regions. The survey included a list of 37 traditional Saudi dishes, and participants were asked to indicate their consumption frequency as: *never*, *less than 3 times per year*, *twice per month*, *four times per month*, *less than 3 times per week (including once per week)*, *or more than 3 times per week*. The objective of the survey was to identify the most commonly consumed Saudi dishes, from which the top 25 dishes were selected for further analysis.

Overall, 360 individuals responded to the survey. From this pool, we identified eligible participants who expressed interest in providing traditional recipes. Inclusion criteria required recipe contributors to be Saudi citizens aged 18–65 years, fluent in Arabic, and of either sex. In addition, participants needed sufficient experience preparing the dish, defined as having cooked it at least five times in the past year. Individuals who did not meet these criteria were excluded.

Ethical approval for all aspects of the survey and recruitment procedures were obtained from the Institutional Review Board of Fakeeh College for Medical Sciences (approval number 686/IRB/2024). To obtain informed consent, written messages were sent via WhatsApp to participants who agreed to be contacted to provide traditional recipes after completing the survey. These messages explained the purpose and procedures of the study. Participants confirmed their consent via WhatsApp (Meta, Menlo Park, CA, USA) before telephone interviews. During each call, participants were verbally reminded that participation was voluntary and that they could withdraw at any time. They were also informed that all data would remain confidential. Personal identifiers (e.g., name and contact details) were collected solely for the purpose of distributing gift cards as tokens of appreciation. These identifiers were stored securely, kept strictly confidential, and were not included in the analysis presented in this manuscript.

### 2.3 Recipe collection and nutritional analysis

In Stage 2, dietitians collected 75 recipes from household Saudi individual who are originally from the central, western, eastern, southern, and northern regions of the country (three recipes for each cuisine). Nutritional analysis of the recipes was performed by trained dieticians using ESHA software. Telephone interviews were conducted with participants to gather detailed information on ingredient weights, measurements, and preparation methods. Trained data collectors applied a standardized procedure for ingredient reporting and interview troubleshooting. Ingredients from household recipes were converted to international weights and measures using FAB-based resources. To account for variability and standardize recipe collection across participants, three independent recipes were collected per dish, and the individual ingredient amounts were averaged. To reduce recall and estimation bias, participants were advised to use the Food Amount Booklet (FAB) (see Supplementary material 1), which provides visual aids and reference measures to convert non-standard household estimates (e.g., “a handful,” “a pinch”) into grams or milliliters. Trained clinical dietitians and data collectors guided participants interactively in their native language, clarifying any ambiguities in portion estimation. This approach minimized bias and ensured consistency, with FAB conversions applied uniformly across all recipes. Data collectors were familiar with the main ingredients of the selected Saudi cuisines, which are listed in Table 1 Nutritional profiles were generated, and nutrient values are presented both per 100 g and by portion size. For traditional ingredients not included in the ESHA database (e.g., jameed), nutritional information was obtained directly from product packaging and manually entered into the ESHA software to ensure accurate, product-specific representation in the analysis.

**Table 1:** Types of dishes and main ingredients of selected traditional Saudi dishes.

Dish type	Categories	Dish name	Main ingredients
Savory dishes	Main dish	Sayaddiyah	Fish, rice, onions, oil
	Appetizer	Mantu	White flour, oil, ground beef, onion
	Main dish	Madoos	Rice, lentils, ghee, garlic, onions, olive oil
	Main dish/Appetizer	Kbebah Hail	Grape leaves, meat, rice, garlic, onion, pepper, tomato, green onion, olive oil, potato, carrot, meat broth
	Main dish	Hasawi Rice	Hasawi rice, meat, onion, tomato, beans
	Main dish	Mansaf	Meat, onion, jameed (fermented dried yogurt), yogurt, rice, oil, bread
	Main dish /Appetizer	Mutabbaq	Mutabbaq leaves, eggs, tomato, green onions, leek, parsley, coriander
	Main dish/Appetizer	Jareesh	Chicken, jareesh (crushed wheat), rice, onion, yogurt, milk, starch, ghee
	Main dish	Meat Kabsah	Lamb meat, rice, tomato, tomato paste, onion, ghee, carrots
	Main dish	Temmn	Temmn (rice, typically long grain like basmati), chicken, onion, olive oil, tomato, pumpkin, eggplant, zucchini
	Main dish	Chicken Kabsah	Chicken, rice, tomato paste, tomato, onion, oil
	Appetizer	Shish Barak	White flour, olive oil, grounded lamb meat, yogurt, onion, corn starch, parsley, coriander
	Main dish	Haneeth	Lamb meat, onion, oil, pepper, rice
	Main dish	Mandi	Lamb meat, rice, olive oil
	Main dish	Saleeq	Chicken, rice, olive oil, powdered milk
	Main dish	Margoug	Olive oil, tomato paste, flour, lamb meat, onion, zucchini, carrot, potato, tomato, green beans
	Main dish	Tharid	Lamb thigh, olive oil, onion, tomato, tomato paste, zucchini, pumpkin, bread tortillas
	Main dish	Raqsh	Whole flour, potato, oil, garlic, onion, lamb thighs
Sweet dishes	Desert	Areekah	Whole wheat flour, ghee, dates
	Desert	Southern Aseedah	Whole wheat flour, dates, oil, butter
	Desert	Marasee	Whole wheat flour, sugar, powder milk, oil, honey, butter
	Desert	Hininy	Date, flour, full fat milk, butter, sugar
	Desert	Saqu	Saqu (A starchy flour extracted from the roots of the cassava plant, known as tapioca), sugar, olive oil, pistachio, cardamom
	Desert	Masoub	Whole wheat bread, banana, honey, sugar, cream
	Main dish	Jamriyah	Whole wheat flour, oil, sugar, milk, sesame, dates, ghee, honey

### 2.4 Statistical analysis

Data are expressed as mean ± standard deviation (SD) to report the nutritional profiles of the recipes. ESHA software was used to generate nutrient composition data per 100 g, with each recipe analyzed in triplicate. The resulting data were exported to Microsoft Excel (Microsoft, Redmond, WA, USA), where means and SD were calculated.

## 3 Results

### 3.1 Participants’ demographic data

A total of 360 individuals participated in the survey. Most respondent were aged 31–45 years (38.9%), whereas the smallest proportion was older than 60 years (4.4%) (Table 2). The majority of participants were women (75.6%). By geographic distribution, the western region contributed the largest proportion (53.6%), while the northern region contributed the smallest (8.3%); 1.7% reported residing in multiple regions. Most respondents were married (65.8%). Overall, the demographic profile represented a heterogeneous cohort but was predominantly composed of middle-aged, married women, with a notable concentration from the western region. Of these respondents, 61 recipe providers provided complete recipe data during telephone interviews.

**Table 2:** Characteristics of dish consumers and recipe providers.

Characteristic	Dish consumers		Recipe providers	
	*n*	%	*n*	%
Total (*N*)	360		61	
Age (years)				
<30	83	23.1	9	14.8
31–45	140	38.9	27	44.3
46–60	121	33.6	22	36.1
>60	16	4.4	3	4.9
Sex				
Female	272	75.6%	50	82.0
Male	88	24.4%	11	18.0
Region				
Southern	37	10.3%	4	6.6
Eastern	46	12.8%	9	14.8
Northern	30	8.3%	10	16.4
Western	193	53.6%	31	50.8
Central	48	13.3%	6	9.8
More than one region	6	1.7%	1	1.6
Marital status				
Single	94	26.1%	10	16.4
Married	237	65.8%	46	75.4
Divorced	29	8.1%	5	8.2

### 3.2 Selected dishes, regional representation, and main ingredients

A total of 25 commonly-consumed traditional Saudi dishes were analyzed. The dishes and main ingredients, grouped by regions, are presented in Table 2.

1. Central region: Haneeth, Jareesh, Hininy, Marasee.
2. Western region: Shish Barak, Meat Kabsah, Saleeq, Mutabbaq, Madoos, Masoub, Mantu, Sayaddiyah, Chicken Kabsah, Tharid, and Mandi.
3. Eastern region: Sagu, Hasawi rice.
4. Southern region: Areekah, Southern Aseedah, Raqsh.
5. Northern region: Margoug, Temmn, Kbebah hail, Mansaf, Jamriyah.

### 3.3 Macronutrient composition of the selected traditional Saudi dishes based on ESHA software analysis

The ESHA analysis revealed variations in the macronutrient and moisture content of the studied dishes, as summarized in Table 3. To facilitate comparison, dishes are presented in descending order of their energy content per 100 g. Areekah, a sweet dish with high carbohydrate (50.16 g) and fat (10.28 g) content, was the most energy-dense dish, whereas Margoug, a savory main dish with high moisture content (80.44 g), was the least energy-dense. The higher energy value of Areekah compared with Margoug reflects differences in their observed carbohydrate, fat, and protein composition. Raqsh had the lowest carbohydrate (5.9 g/100 g), while Haneeth as a main dish contained the lowest fat content (1.96 g/100 g). Fiber content also varied widely: Southern Aseedah and Areekah showed the highest levels (5.83 g and 5.67 g, respectively), whereas Saleeq and Shish Barak had the lowest (0.26 g and 0.53 g, respectively). Protein content was highest in Haneeth (12.96 g) and Mansaf (12.27 g), consistent with their meat-based composition.

**Table 3:** Macronutrients composition of the 25 Saudi dish recipes based on ESHA software analysis.

No.	Dish recipe	Energy (kcal)	Protein (g)	Carbohydrate (g)	Total fiber (g)	Fat (g)	Water (g)
1.	Areekah	306.86 ± 8.27	5.63 ± 0.12	50.16 ± 2.63	5.67 ± 1.36	10.28 ± 1.04	31.17 ± 1.36
2.	Southern Aseedah	251.58 ± 70.53	4.24 ± 3.99	48.22 ± 18.71	5.83 ± 4.15	6.09 ± 7.25	23.46 ± 2.22
3.	Saqu	249.6 ± 32.8	9.91 ± 3.14	37.1 ± 3.99	1.27 ± 0.36	7.03 ± 4.13	48.88 ± 5.1
4.	Jamriyah	242.5 ± 66.48	5.24 ± 1.36	35.67 ± 6.13	4.14 ± 1.89	9.65 ± 7.09	45.79 ± 9.9
5.	Mansaf	239.86 ± 31.58	12.27 ± 1.09	16.25 ± 3.38	0.56 ± 0.17	13.25 ± 3.87	36.47 ± 8.45
6.	Marasee	230.91 ± 85.72	7.26 ± 2.22	41.09 ± 16.26	3.95 ± 0.93	4.49 ± 0.76	5.74 ± 2.27
7.	Hininy	196.22 ± 29.72	3.43 ± 1.06	37.52 ± 4.33	2.8 ± 1.17	5.06 ± 1.82	7.05 ± 3.28
8.	Masoob	180.47 ± 21.53	3.79 ± 0.63	35.15 ± 4.59	4.7 ± 1.61	4.05 ± 0.47	44.39 ± 9.1
9.	Sayaddiyah	172.52 ± 93.46	9.38 ± 4.1	26.61 ± 21.45	1.33 ± 0.8	2.76 ± 2.92	30.7 ± 26.34
10.	Mantu	160.7 ± 74.21	6.29 ± 3.09	21.36 ± 7.03	1.3 ± 0.51	5.35 ± 4.46	55.71 ± 24.44
11.	Mutabbaq	156.59 ± 35.54	7.01 ± 1.2	15.95 ± 3.32	1.95 ± 0.56	6.94 ± 2.88	56.2 ± 6.65
12.	Meat Kabsah	137.41 ± 17.04	7.28 ± 0.77	15.56 ± 4.43	0.72 ± 0.13	4.97 ± 0.38	68.88 ± 4.96
13.	Hasawi Rice	134.96 ± 21	7.08 ± 1.25	16.97 ± 3.9	1.76 ± 0.42	4.45 ± 0.53	39.53 ± 7.54
14.	Kbebah Hail	127.08 ± 18.12	5.01 ± 2.85	18.01 ± 4.2	1.65 ± 0.16	4.07 ± 1.8	37.1 ± 9.3
15.	Shish Barak	126.28 ± 49.33	4.63 ± 0.82	13.83 ± 7.22	0.53 ± 0.26	5.43 ± 1.39	32.87 ± 17.22
16.	Madoos	126.17 ± 22.56	3.99 ± 1.04	18.34 ± 4.86	1.57 ± 0.26	4.14 ± 0.32	71.14 ± 6.26
17.	Haneeth	125.47 ± 3.59	12.96 ± 0.51	12.45 ± 1.2	0.37 ± 0.02	1.96 ± 0.61	44.36 ± 3.08
18.	Jareesh	119.65 ± 15.26	5.06 ± 0.65	15.72 ± 4.59	0.75 ± 0.11	3.92 ± 1.54	22.32 ± 11.53
19.	Temmn	117.71 ± 21.1	5.71 ± 1.07	14.67 ± 3.16	0.71 ± 0.22	4.03 ± 0.63	44.59 ± 7.78
20.	Raqsh	115.59 ± 6.82	7.17 ± 1.73	5.9 ± 1.89	1.06 ± 0.35	7.19 ± 0.21	79.36 ± 1.44
21.	Saleeq	111.81 ± 15.4	6.51 ± 0.99	11.08 ± 3.39	0.26 ± 0.19	4.32 ± 2.2	74.6 ± 2.94
22.	Chicken Kabsah	108.43 ± 20.92	5.06 ± 1.24	14.64 ± 1.14	0.68 ± 0.11	3.26 ± 1.18	74.54 ± 3.58
23.	Mandi	104.27 ± 27.19	5.42 ± 2.05	11.15 ± 4.83	0.33 ± 0.14	3.9 ± 2.37	77.33 ± 5.29
24.	Tharid	99.47 ± 13.28	5.83 ± 1.44	9.24 ± 0.69	0.82 ± 0.13	4.53 ± 1.08	78.69 ± 1.98
25.	Margoug	89.21 ± 19.2	4.08 ± 0.62	10.98 ± 0.73	1.82 ± 0.22	3.6 ± 1.61	80.44 ± 2.91

Data represent mean ± SD of the three recipes per dish. Dishes are ordered from the highest to lowest energy per 100 g.

### 3.4 Macronutrient energy distribution of the selected traditional Saudi dishes based on ESHA software analysis

**Figure 1:**
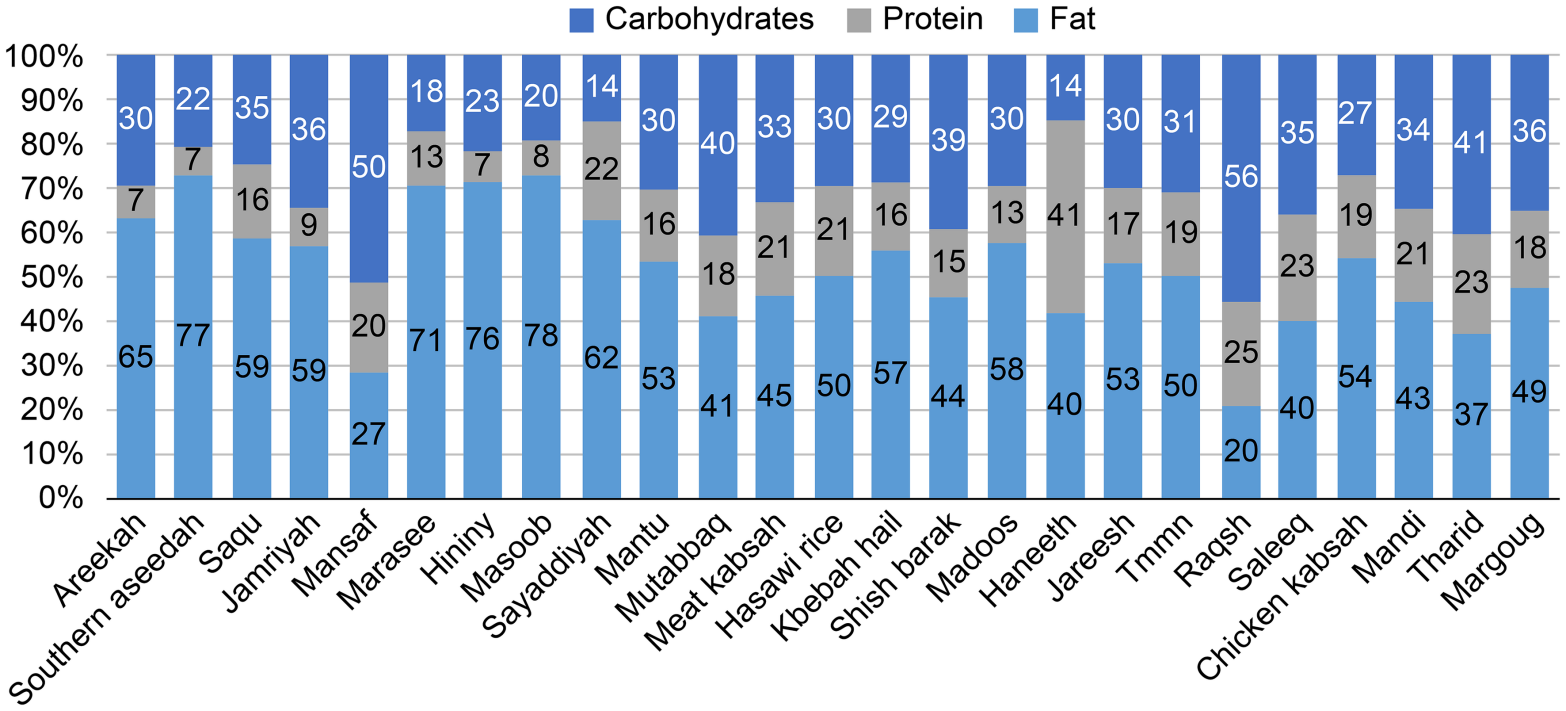
Percentage distribution of energy from carbohydrate, protein, and fat in 25 traditional Saudi dish recipes. Values represent the proportion of total energy contributed by each macronutrient. Dishes are arranged from left to right in descending order of energy content per 100 g.

The contribution of dietary energy varied across the traditional dishes analyzed (Figure 1). Overall, the dishes provided 20–78% of the total energy from carbohydrates, 7–41% from protein, and 14–56% from fat. Carbohydrates accounted for more than half of the total energy in 15 dishes. In contrast, fat contributed over 50% of the total energy in only one dish—Raqsh—which derived 56% of its energy from fat and only 20% from carbohydrates. Conversely, Masoob derived the majority of its energy from carbohydrates (78%), with only 20% from fat. Protein contribution ranged from as low as 7% in Areekah, Southern Aseedah, and Hininy, to as high as 25% in Raqsh.

### 3.5 Sugar, fat classification, and sodium levels in the selected traditional Saudi dishes based on ESHA software analysis

Table 4 presents the sugar content of the selected dishes. Among the dishes, Saqu (22.12 g/100 g) and Hininy (21.62 g/100 g) had the highest observed sugar contents, whereas Mandi (0.03 g/100 g) and Haneeth (0.05 g/100 g) had the lowest observed values.

**Table 4:** Sugar, fat types, and sodium content of traditional food recipes based on ESHA software analysis.

N	Dish recipe	Sugar (g)	Added sugar (g)	Saturated fat (g)	Monounsaturated fat (g)	Polyunsaturated fat (g)	Trans fat (g)	Cholesterol (mg)	Sodium (mg)
1.	Areekah	6.12 ± 6.12	3.24 ± 3.42	5.82 ± 0.59	0.12 ± 0.03	0.51 ± 0.12	0 ± 0.59	18.76 ± 1.93	106.17 ± 97.2
2.	Southern Aseedah	20.14 ± 20.14	0.36 ± 0	2.00 ± 1.91	1.07 ± 2.22	1.93 ± 3.42	0.03 ± 1.91	5.25 ± 5.15	140.31 ± 88.4
3.	Saqu	22.12 ± 22.12	19.49 ± 2.04	0.95 ± 0.55	1.59 ± 1.19	2.66 ± 2.34	0 ± 0.55	18.49 ± 6.01	278.6 ± 89.7
4.	Jamriyah	14.51 ± 14.51	7.19 ± 2.4	3.64 ± 2.63	1.22 ± 1.43	2.50 ± 2.70	0 ± 2.63	12.26 ± 9.8	225.78 ± 63.43
5.	Mansaf	0.53 ± 0.53	0.04 ± 0.01	5.25 ± 1.69	4.54 ± 1.25	1.71 ± 1.12	0.26 ± 1.69	39.47 ± 10.37	135.65 ± 74.96
6.	Marasee	8.18 ± 8.18	5.35 ± 3.17	1.60 ± 0.59	1.09 ± 0.73	1.01 ± 0.24	0 ± 0.59	41.52 ± 30.22	588.19 ± 144.52
7.	Hininy	21.62 ± 21.62	0.61 ± 1.06	2.91 ± 1.05	0.71 ± 0.08	0.15 ± 0.07	0.11 ± 1.05	12.19 ± 4.07	107.27 ± 104.18
8.	Masoob	9.72 ± 9.72	3.39 ± 4.13	2.13 ± 0.57	0.34 ± 0.48	0.33 ± 0.25	0.05 ± 0.57	8.53 ± 2.91	79.54 ± 42.13
9.	Sayaddiyah	1.65 ± 1.65	0 ± 0	0.44 ± 0.43	1.12 ± 1.41	0.78 ± 0.86	0 ± 0.43	26.42 ± 11.28	219.12 ± 98.6
10.	Mantu	1.44 ± 1.44	0 ± 0	0.29 ± 0.19	0.39 ± 0.21	0.48 ± 0.2	0.02 ± 0.19	1.39 ± 2.41	207.88 ± 36.89
11.	Mutabbaq	1.31 ± 1.31	0 ± 0	1.1 ± 0.55	1.63 ± 0.63	1.31 ± 1.31	0.05 ± 0.55	81.12 ± 19.87	281.67 ± 45.23
12.	Meat Kabsah	0.79 ± 0.79	0 ± 0	2.12 ± 0.18	0.1 ± 0.06	0.20 ± 0.10	0.28 ± 0.18	22.41 ± 2.61	145.65 ± 58.29
13.	Hasawi Rice	1.33 ± 1.33	0 ± 0	1.88 ± 0.43	0.25 ± 0.22	0.17 ± 0.19	0.24 ± 0.43	17.89 ± 1.93	257.65 ± 104.81
14.	Kbebah Hail	1.59 ± 1.59	0 ± 0	1.23 ± 1.18	2.14 ± 0.55	0.44 ± 0.08	0.13 ± 1.18	8.13 ± 11.05	197.5 ± 40.94
15.	Shish Barak	2.87 ± 2.87	0 ± 0	0.91 ± 0.27	0.56 ± 0.19	0.51 ± 0.68	0 ± 0.27	7.78 ± 3.95	202.58 ± 33.66
16.	Madoos	0.55 ± 0.55	0 ± 0	1.71 ± 0.18	1.13 ± 0.32	0.23 ± 0.06	0 ± 0.18	4.91 ± 0.73	174.6 ± 18.07
17.	Haneeth	0.05 ± 0.05	0 ± 0	0.48 ± 0.10	0.74 ± 0.19	0.38 ± 0.30	0 ± 0.1	32.64 ± 1.71	290.28 ± 80.14
18.	Jareesh	1.44 ± 1.44	0 ± 0	1.62 ± 0.44	0.7 ± 1.1	0.35 ± 0.51	0.06 ± 0.44	15.09 ± 6.62	196.76 ± 96.83
19.	Temmn	1.14 ± 1.14	0 ± 0	1.50 ± 0.58	0.71 ± 1.08	0.37 ± 0.38	0.16 ± 0.58	17.17 ± 3.96	214.51 ± 70.35
20.	Raqsh	0.35 ± 0.35	0 ± 0	2.63 ± 0.19	0.98 ± 0.85	0.76 ± 0.32	0.32 ± 0.19	25.21 ± 6.21	87.33 ± 31.44
21.	Saleeq	0.67 ± 0.67	0 ± 0	1.54 ± 0.71	1.51 ± 0.9	0.68 ± 0.38	0.04 ± 0.71	22.54 ± 5.29	89.16 ± 28.53
22.	Chicken Kabsah	0.94 ± 0.94	0 ± 0	0.82 ± 0.44	1.45 ± 0.4	0.65 ± 0.27	0.01 ± 0.44	13.86 ± 5.63	100.48 ± 19.01
23.	Mandi	0.03 ± 0.03	0 ± 0	1.53 ± 0.85	0.43 ± 0.53	0.06 ± 0.08	0.22 ± 0.85	16.8 ± 8.73	144.2 ± 14.84
24.	Tharid	1.46 ± 1.46	0 ± 0	1.66 ± 0.5	0.6 ± 0.3	0.28 ± 0.24	0.22 ± 0.5	17.53 ± 5.88	92.63 ± 12.11
25.	Margoug	1.2 ± 1.2	0 ± 0	1.15 ± 0.27	1.01 ± 1.02	0.27 ± 0.13	0.13 ± 0.27	9.94 ± 1.23	184.58 ± 43.97

The fat composition of each dish, including healthy (monounsaturated and polyunsaturated) and unhealthy fats (trans and saturated fats), was also analyzed. Areekah (5.82 g) and Mansaf (5.25 g) contained the highest amounts of saturated fat, while Mantu (0.29 g) and Sayaddiyah (0.44 g) contained the lowest. Trace amounts of trans fat were detected across all dishes (>0 but <0.5 g per 100 g, as defined by the ESHA nutrient analysis software).

Sodium content varied widely among the dishes. The lowest sodium level was reported in Masoob, while the highest was in Marasee. Seven dishes—including Masoob, Areekah, Hininy, Raqsh, Saleeq, Chicken Kabsah, and Tharid—had sodium levels below 140 mg/100 g and may be classified as low-sodium dishes. Further micronutrients content of the traditional recipes is provided in Supplementary Tables S1–S3.

These findings highlight the diversity of nutritional profiles across traditional Saudi dishes and provide insights for dietary planning and nutritional assessments in the region.

### 3.6 Amount of the dish recipes containing 15 g of carbohydrates in the selected traditional Saudi dishes based on ESHA software analysis

The weights of various traditional dishes recipes required to supply 15 g of available carbohydrate were estimated (Table 5). Among the most carbohydrate-dense dishes were Areekah, Southern Aaseesidah, Maraseeyaa, Hianinyi, Saqu, Jamriyah, and Masoob, respectively. Less than 50 g was required to provide one exchange list of carbohydrate (15 g). On the other hand, the least carbohydrate-dense were Threed, Margougqoq, Saleeq, and Raqsh; large portions are required to provide the 15 g of carbohydrate.

**Table 5:** Amount of the dish recipes containing 15 g of carbohydrates.

Dish recipe	Weight of each dish containing 15 g carbohydrate
Areekah	29.90
Southern Aseedah	31.11
Marasee	36.51
Hininy	39.98
Saqu	40.43
Jamriyah	42.05
Masoob	42.67
Sayaddiyah	56.37
Mantu	70.22
Madoos	81.77
Kbebah Hail	83.27
Hasawi	88.39
Mansaf	92.33
Mutabbaq	94.06
Jareesh	95.45
Meat Kabsah	96.39
Temmn	102.25
Chicken Kabsah	102.46
Shish Barak	108.43
Haneeth	120.48
Mandi	134.53
Saleeq	135.38
Margoug	136.65
Threed	162.28
Raqsh	254.38

## 4 Discussion

This study analyzed the nutritional profiles of 25 most commonly consumed traditional Saudi dishes and provides their detailed composition. Dishes were selected through a questionnaire distributed across the five regions of Saudi Arabia, with the largest proportion of participants (53.6%) from the western region. The results revealed notable differences in the nutritional composition of the selected dishes. Ten dishes had moisture levels exceeding 50%. Margoug exhibited the highest moisture content (80.4%), whereas Marasee had the lowest (5.7%). Haneeth contained the highest protein content (12.9%), while Hinini had the lowest (3.4%). These findings suggest that some dishes can serve as important sources of protein, while others may need to be paired with additional foods to meet daily protein requirements. Fat content ranged from 1.9% in Haneeth to 13.3% in Mansaf, supporting higher energy intake. Fiber content varied widely, with Southern Aseedah highest (5.8%) and Saleeq having the lowest content (0.26%). Carbohydrate content ranged from 50.1% in Areekah to 5.9% in Raqsh, offering options to accommodate different energy needs and dietary preferences.

Previous studies examining the nutritional composition of traditional Saudi dishes primarily relied on laboratory-based proximate analyses. For example, Al-Kanhal et al. analyzed wheat-based dishes such as Margoug, Marasee, Jareesh, and Harees (9), as well as rice-based dishes such as Kabsah (11). Similarly, Al-Faris (12) reported laboratory results for several traditional dishes, including Mutabbaq, Margoug, Aseedah, and Meat Kabsah. The present work overlaps with six of these dishes—Mutabbaq, Marasee, Southern Aseedah, Margoug, Jareesh, and Meat Kabsah—and expands the dataset by including an additional 19 dishes. Protein values in our study were largely similar to those previously reported (12). In contrast, carbohydrate content was higher in Mutabbaq, Margoug, and Meat Kabsah in the study by Al-Faris, whereas Aseedah showed lower carbohydrate value. Fat content was lower in Aseedah and Margoug but higher in Meat Kabsah compared with our data. Fiber content in Marasee and Mutabbaq was also lower in prior studies, while water content in Aseedah and Marasee was notably higher (12). More recently, Mir et al. (14) evaluated 25 commonly consumed Saudi dishes, eight of which overlapped with our analysis (Aseedah, Marasee, Sayaddiyah, Meat Kabsah, Jareesh, Saleeq, Mandi, and Margoug). Their reported protein values were similar to ours for most dishes, except Meat Kabsah, which showed nearly half the protein content observed in our study. Fat content in our samples was two- to three-fold higher for Meat Kabsah, Aseedah, Jareesh, Marasee, and Saleeq, while Sayaddiyah showed twice the fat content compared with their data. Carbohydrate content was generally consistent across studies, except for Aseedah (three-fold higher in our data) and Saleeq (five-fold lower). These discrepancies highlight potential differences that may arise when comparing database-derived estimates with direct laboratory analyses; thus, they should be interpreted as contextual rather than definitive.

It is important to acknowledge the methodological differences across studies. Al-Kanhal et al. (9–11) and Al-Faris (12) relied on laboratory-based chemical analyses, while Mir et al. (14) combined laboratory evaluations with dietary assessment tools. In contrast, our nutrient composition values were estimated using the ESHA Food Processor software, which calculates nutrient values based on food composition databases and recipe input. Unlike laboratory methods, ESHA does not account for nutrient changes due to cooking time, processing losses, or anti-nutritional factors. However, software-based analysis offers efficiency, scalability, and the ability to capture variations in ingredient proportions across recipes, making it a practical alternative to lengthy laboratory procedures. These methodological differences likely explain some of the variability observed across studies. Laboratory approaches provide precise measurements under controlled conditions but are resource-intensive and limited in scope. In contrast, software-based estimation, as used in the present study, allows for broader coverage of recipes and standardization across datasets, albeit with some inherent limitations. Therefore, cross-study comparisons should be interpreted cautiously. Future research should aim to standardize assessment methods to minimize variability and ensure greater comparability across studies.

This study also analyzed the dietary energy and nutrient composition of 25 commonly-consumed traditional Saudi dishes. Energy content ranged from 89.2 kcal in Margoug to 306.8 kcal in Areekah, with the latter representing an energy-dense option suitable for individuals with high caloric needs (Table 3). According to the British Nutrition Foundation, foods with very low, low, medium, and high energy density contain <0.6, 0.5–2.0, 1.7–5.0, and >5 kcal/g, respectively (15). Based on this classification, 15 dishes were categorized as low-energy-density foods and 10 as high-energy-density foods. Dishes with lower energy density, such as Margoug, Tharid, and Mandi, supply fewer calories per gram, allowing larger portions with low caloric intake and supporting their role in weight management (16).

Carbohydrate content varied widely, with the highest levels observed in sweet dishes, including Areekah, Southern Aseedah, Marasee, Hininy, Saqu, and Masoob and lower levels in savory dishes. Excessive carbohydrate consumption is associated with an increased risk of type 2 diabetes and related complications (17). Thus, high-carbohydrate dishes should be consumed in moderation. The quality of carbohydrates is also important: dishes incorporating whole grains, legumes, and vegetables (e.g., Temmn, Saleeq, Margoug, Kbebah Hail, Jareesh, Mutabbaq, Sayaddiyah) are healthy “good carb” options compared with those made with refined sugars and processed flours.

The macronutrient distribution of the dishes was assessed according to the acceptable macronutrient distribution range established by the U. S. Food and Nutrition Board (45–65% carbohydrates, 10–35% protein, and 20–35% fat) (Food & Nutrition Board, Institute of Medicine, 2006) (18). Most dishes provided balanced proportions of macronutrients. However, 16 dishes contributed <45 of energy from carbohydrates, while four dishes—Masoob, Southern Aseedah, Hininy, and Marasee—approached the upper carbohydrate limit (65%). Protein content was <10 in 20 dishes, indicating low protein density. Fat exceeded 35% of energy in six dishes (Raqsh, Mansaf, Tharid, Mutabbaq, Shish Barak, and Margoug), which may contribute to weight gain and chronic disease risk. These results should be interpreted as approximate database-derived estimates, as nutrients can vary with preparation methods and ingredients.

This study provides the first overview of the estimated lipid profile of traditional Saudi foods, including saturated, monounsaturated, polyunsaturated, and trans fats derived from ESHA analysis. According to international recommendations saturated fat should not exceed 10% of total energy intake (<20 g/day in a 2,000-calorie diet), and trans fat should remain <1% (<2.2 g/day) (19). Areekah and Mansaf exceeded the high saturated fat threshold (>5 g/100 g), warranting moderation.

In contrast, Sayaddiyah, Mantu, and Chicken Kabsah contained <0.5 g/100 g. Trans fats were detected in trace amounts (<0.5 g/100 g) across the recipes analyzed, remaining below international thresholds.

Sodium content was also variable. World Health Organization guidelines recommend limiting sodium intake to <2,000 mg/day, with <140 mg/serving classified as low sodium and >500 mg high (16). Marasee was the only high-sodium dish (588.19 mg/100 g). Seven dishes (Masoob, Areekah, Hininy, Raqsh, Saleeq, Chicken Kabsah, Tharid) qualified as low-sodium options, while the remainder fell in the moderate range. This is particularly relevant for individuals with hypertension or cardiovascular disease.

Dietary patterns strongly influence health outcomes, particularly obesity, diabetes, and cardiovascular disease. Previous studies indicate low adherence to dietary guidelines among Saudis, with high fat intake and low consumption of fish, nuts, and vegetables (4, 20). Although traditional dishes provide valuable nutrients, they may also be energy-dense and variable in fat and sugar content, particularly with the addition of ingredients such as honey, butter, or ghee. These variations highlight the importance of portion control and mindful consumption.

### 4.1 Strength and limitations

This study analyzed the nutritional composition of 25 commonly-consumed traditional Saudi dishes using ESHA software, providing standarized and comparable data. The ESHA database allows for customized entries of traditional ingredients such as ground cumin in Mandi and jameed in Mansaf, thereby generating culturally relevant nutrient profiles. These results can inform research, education, and national dietary guidelines.

However, some limitations should be noted. First, ESHA estimates are based on raw ingredient weights, which may not fully reflect post-cooking nutrient values, as cooking time and temperature can alter nutrient levels. Second, missing local ingredients (e.g., homemade flours) required manual substitutions, necessitating further verification to ensure accuracy. Third, estimating ingredient quantities was challenging for some participants who relied on non-standard measures (e.g., “a handful”), although the food amount booklet helped reduce misreporting. Herbs and spices used in small quantities (e.g., stone flower) were also difficult to quantify precisely. In addition, the reliance on self-reported recipes introduced recall bias, while regional variations in ingredients and preparation limited the ability to define standard nutritional profiles. Finally, participant health status and household dietary practices (e.g., salt, sugar, or fat adjustments for chronic illness) were not taken into consideration, which may have influenced recipe composition. Despite these limitations, steps were taken to improve accuracy, including manual data verification, careful selection of substitutes, and averaging three recipes per dish. These measures strengthened the reliability and representativeness of the findings.

### 4.2 Implications and future directions

Understanding the nutritional composition of traditional dishes supports healthier dietary choices and provides evidence for public health strategies aimed at addressing type 2 diabetes, obesity, and cardiovascular diseases. Nutritional profiles can guide nutritionists and healthcare professionals in developing culturally-appropriate dietary recommendations and exchange lists, while also informing updates to dietary guidelines that respect cultural preferences. These findings may further influence the food industry to adapt traditional recipes into healthier versions and support policy initiatives on food labeling and nutrition education.

In addition, future research should focus on laboratory validation of ESHA-derived estimates and include assessment of the glycemic index of these dishes, particularly for diabetes management to ensure greater accuracy and applicability of the findings. Developing an exchange list of traditional Saudi foods could benefit both individuals and clinicians by enabling culturally-relevant substitutions without altering energy or macronutrient intake, thereby facilitating tailored meal plans for both healthy individuals and those with obesity-related diseases or diabetes (21). While recipe and preparation variability in this study precluded standardized serving sizes or average exchange lists, values could be estimated using the American Dietetic Association and American Diabetes Association exchange systems (22). Adapting traditional recipes to meet modern nutritional needs while preserving cultural identity remains essential. Tailored dietary interventions that incorporate modified versions of traditional dishes should be explored for individuals with diabetes and metabolic syndrome. In addition, technology-based tools—such as mobile apps and online databases—could enhance the dissemination of nutritional information, making it more accessible to the public and healthcare providers alike.

## Data Availability

The raw data supporting the conclusions of this article will be made available by the authors, without undue reservation.
