# Supplementary material for: Comprehensive nutritional compositions of traditional dishes from five Saudi regions using Elizabeth Stewart Hands and Associates Food Processor software
Source: Front Nutr. 2025 Nov 10;12:1658078. doi: 10.3389/fnut.2025.1658078 (PMC12641437; doi:10.3389/fnut.2025.1658078)
Supplement: Supplementary file 1 [file Table_1.DOCX]

Supplemental Table 1

Water-soluble vitamins composition of the 25 Saudi dish recipes based on ESHA software analysis.

| **Recipe** | **Vit B1 (mg)** | **Vit B2 (mg)** | **Vit B3 (mg)** | **Vit B3-NE (mg)** | **Vit B6 (mg)** | **Vit B12 (mcg)** | **Biotin (mcg)** | **Vit C (mg)** | **Folate (mcg)** | **Fol-DFE (mcg DFE)** | **Pantothenic acid (mg)** |
| --- | --- | --- | --- | --- | --- | --- | --- | --- | --- | --- | --- |
| **AREEKAH** | 0.26±0.08 | 0.16±0.05 | 3.08±0.12 | 3.47±0.46 | 0.08±0.02 | 0±0 | 0.29±0.01 | 0.02±0.02 | 44.41±25.09 | 64.47±42.46 | 0.46±0.09 |
| **SOUTHERN ASSIDAH** | 0.17±0.1 | 0.16±0.17 | 2.31±1.82 | 2.5±1.65 | 0.12±0.04 | 0±0.01 | 0.16±0.08 | 0.15±0.31 | 25.41±13.16 | 33.76±23.82 | 0.51±0.27 |
| **Saqu** | 0.01±0 | 0.01±0 | 0.02±0.01 | 0.06±0.02 | 0.02±0.01 | 0±0 | #DIV/0! | 0.07±0.02 | 0.62±0.15 | 0.62±0.15 | 0.01±0.01 |
| **Jamriah** | 0.15±0.03 | 0.18±0.1 | 2.18±0.3 | 1.45±0.62 | 0.12±0.04 | 0.32±0.28 | 1.9±1.68 | 9.76±8.57 | 7.63±3.24 | 7.63±3.24 | 0.27±0.12 |
| **MANSAF** | 0.09±0.04 | 0.15±0 | 3.64±0.63 | 5.17±0.96 | 0.16±0.05 | 0.93±0.61 | 0.43±0.35 | 0.62±0.27 | 6.48±5.96 | 6.48±5.96 | 0.34±0.11 |
| **MARASYAA** | 0.49±0.31 | 0.5±0.32 | 4.1±1.52 | 4.13±1.98 | 0.34±0.42 | 0.98±0.9 | 1.32±0.71 | 5.99±3.78 | 69.74±61.46 | 67.72±101.16 | 0.34±0.06 |
| **HANINI** | 0.03±0.03 | 0.03±0.03 | 0.71±0.56 | 0.7±0.5 | 0.08±0.05 | 0.03±0.04 | #DIV/0! | 0.73±1.26 | 5.42±3.19 | 5.42±3.19 | 0.27±0.14 |
| **MASOOB** | 0.08±0.06 | 0.08±0.04 | 1.47±1.03 | 1.54±1.04 | 0.21±0.04 | 0±0 | 1.18±0.15 | 4.01±0.44 | 15.22±5.28 | 15.22±5.28 | 0.38±0.19 |
| **SAYADIAH** | 0.24±0.18 | 0.11±0.09 | 3.5±1.98 | 2.87±2.4 | 0.14±0.14 | 0.58±0.14 | 0.58±0.28 | 1.2±0.34 | 23.08±27.55 | 30.63±44.79 | 0.26±0.34 |
| **MANTU** | 0.21±0.06 | 0.14±0.04 | 1.66±0.29 | 2.34±0.34 | 0.07±0.02 | 0.06±0.11 | 1.34±0.44 | 3.38±2.49 | 51±17.49 | 76.97±26.06 | 0.17±0.03 |
| **MUTABBAQ** | 0.08±0.07 | 0.15±0.02 | 0.87±0.5 | 1.75±0.56 | 0.12±0.04 | 0.3±0.19 | 4.86±1.48 | 7.94±4.02 | 38.18±14.99 | 44.66±25.52 | 0.42±0.07 |
| **Meat Kabsah** | 0.13±0.03 | 0.16±0.03 | 3.2±1.06 | 2.07±0.23 | 0.13±0.02 | 0.78±0.09 | 0.59±0.38 | 3.1±1.67 | 2.78±1.28 | 2.78±1.28 | 0.17±0.02 |
| **HASAWI** | 0.06±0.03 | 0.11±0.02 | 1.59±0.21 | 1.72±0.31 | 0.12±0.02 | 0.57±0.08 | 0.8±0.22 | 4.59±1.36 | 15.5±13.73 | 15.5±13.73 | 0.17±0.04 |
| **KBEBAH HAIL** | 0.03±0.02 | 0.08±0.05 | 1.18±1 | 1.64±1.59 | 0.13±0.07 | 0.1±0.15 | 0.66±0.19 | 10.7±7.37 | 18.66±8.04 | 18.66±8.04 | 0.12±0.09 |
| **SHISH BARAK** | 0.11±0.07 | 0.08±0.04 | 0.84±0.51 | 1.17±0.7 | 0.02±0.01 | 0.01±0.01 | 0.5±0.31 | 1.3±0.16 | 27.97±15.99 | 42.56±25.1 | 0.1±0.06 |
| **Madoos** | 0.13±0 | 0.03±0 | 0.62±0.39 | 0.82±0.22 | 0.08±0.02 | 0±0 | 0.24±0.02 | 1.2±0.23 | 60.26±16.96 | 60.26±16.96 | 0.28±0.08 |
| **HANEETH** | 0.16±0.01 | 0.3±0.02 | 3.72±0.06 | 5.07±0.27 | 0±0 | 0.65±0.03 | 0.04±0.04 | 0.22±0.33 | 3.1±0.38 | 3.1±0.38 | 0±0 |
| **JAREESH** | 0.02±0.01 | 0.04±0.01 | 1.14±0.58 | 1.65±1.07 | 0.09±0.04 | 0.11±0.13 | 0.48±0.25 | 1.54±1.13 | 3.6±1.99 | 3.6±1.99 | 0.17±0.1 |
| **TMMN** | 0.05±0.01 | 0.09±0.05 | 1.76±0.15 | 2.06±0.43 | 0.13±0 | 0.44±0.34 | 0.53±0.13 | 6.12±3.23 | 8.68±3.78 | 8.68±3.78 | 0.22±0.07 |
| **Raqsh** | 0.08±0.01 | 0.16±0.04 | 2.51±0.54 | 2.53±0.54 | 0.15±0.03 | 0.87±0.26 | 0.27±0.18 | 1.12±0.35 | 3.34±1.18 | 3.34±1.18 | 0.24±0.04 |
| **SALEEQ** | 0.06±0.06 | 0.06±0.01 | 2.65±0.61 | 2.99±0.8 | 0.11±0.03 | 0.12±0.03 | 0.28±0.1 | 1.79±0.31 | 2.3±1.04 | 2.3±1.04 | 0.26±0.07 |
| **Chicken Kabsah** | 0.07±0.04 | 0.04±0.02 | 2.11±1.14 | 2.26±0.52 | 0.11±0.01 | 0.05±0.03 | 0.7±0.12 | 2.86±0.47 | 4.31±0.51 | 4.31±0.51 | 0.21±0.04 |
| **MANDI** | 0.12±0.03 | 0.12±0.05 | 2.86±0.64 | 1.46±0.76 | 0.08±0.05 | 0.6±0.32 | 0.03±0.05 | 0.1±0.11 | 0.2±0.27 | 0.2±0.27 | 0.11±0.06 |
| **Threed** | 0.06±0.01 | 0.12±0.04 | 1.85±0.45 | 1.95±0.43 | 0.16±0.02 | 0.62±0.21 | 0.36±0.15 | 7.04±1.53 | 9.02±1.84 | 9.02±1.84 | 0.23±0.02 |
| **MARQOQ** | 0.08±0.02 | 0.1±0.03 | 1.69±0.18 | 1.8±0.26 | 0.12±0.03 | 0.35±0.05 | 0.58±0.15 | 5.54±2.01 | 12.25±4.66 | 14.1±7.78 | 0.23±0.01 |

Supplemental Table 2: Fat-soluble vitamins composition of the 25 Saudi dish recipes based on ESHA software analysis.

|  | **Vit A-IU (IU)** | **Vit A-RAE (mcg)** | **Caroten (mcg)** | **Retinol (mcg)** | **BetaCaro (mcg)** | **Vit D (IU)** | **Vit D (mcg)** | **Vit E(mg)** | **Vit K (mcg)** |
| --- | --- | --- | --- | --- | --- | --- | --- | --- | --- |
| **AREEKAH** | 3.33±1.42 | 0.17±0.07 | 0.34±0.14 | 0±0 | 1.85±0.79 | 0±0 | 0±0 | 0.21±0.07 | 0.76±0.25 |
| **SOUTHERN ASSIDAH** | 64.36±59.72 | 7.5±13.1 | 4.47±3.58 | 5.38±12.42 | 26.04±21.24 | 0±0 | 0±0 | 0.84±1.49 | 1.57±0.36 |
| **Saqu** | 6.34±1.49 | 0.32±0.08 | 0.63±0.15 | 0±0 | 3.75±0.89 | 0±0 | 0±0 | 1.1±1.01 | 0.09±0.09 |
| **Jamriah** | 2.44±1.03 | 0.12±0.05 | 0.24±0.1 | 0±0 | 1.35±0.58 | 1.58±1.4 | 0±0 | 1.07±1.15 | 0.59±0.21 |
| **MANSAF** | 5.44±6.88 | 1.55±1.92 | 0.03±0.02 | 0.91±0.9 | 7.68±13.18 | 0.13±0.23 | 0±0.01 | 0.53±0.48 | 2.58±4.25 |
| **MARASYAA** | 126.07±120.8 | 32.45±29.75 | 2.16±3.22 | 31.37±28.54 | 12.79±19.28 | 17.64±14.63 | 0.42±0.38 | 0.43±0.06 | 9.66±15.59 |
| **HANINI** | 124.7±26.88 | 24.5±3.58 | 5.1±2.06 | 22.07±3.02 | 30.55±12.32 | 0.12±0.2 | 0±0 | 0.11±0.05 | 1.26±0.68 |
| **MASOOB** | 31.13±3.71 | 1.56±0.19 | 3.11±0.37 | 0±0 | 12.94±1.59 | 0±0 | 0±0 | 0.21±0.15 | 0.86±0.57 |
| **SAYADIAH** | 102.3±138.06 | 13.95±10.97 | 7.22±14.16 | 10.34±10.45 | 36.61±69.79 | 82.08±163.4 | 2.06±4.1 | 0.43±0.19 | 1.21±0.7 |
| **MANTU** | 212.09±363.26 | 10.6±18.17 | 21.21±36.32 | 0±0 | 107.77±184.33 | 1.45±2.51 | 0.04±0.06 | 0.22±0.09 | 1.06±1.13 |
| **MUTABBAQ** | 659.57±157.26 | 65.3±24.2 | 55.43±13.9 | 31.64±11.47 | 392.72±162.17 | 18.91±5.95 | 0.46±0.15 | 1.1±0.76 | 40.67±37.75 |
| **Meat Kabsah** | 185.95±78.93 | 9.41±3.85 | 18.55±7.9 | 0±0 | 99.84±41.51 | 0.84±0.1 | 0.02±0 | 0.24±0.01 | 1.21±0.7 |
| **HASAWI** | 866.72±1103.45 | 49.11±62.56 | 84.71±107.99 | 5.89±7.17 | 364.24±524.23 | 0.62±0.08 | 0.01±0.01 | 0.26±0.16 | 5.77±7.01 |
| **KBEBAH HAIL** | 3108.73±540.19 | 171.36±33.4 | 310.74±54.16 | 0.46±0.65 | 1985.59±391.54 | 0±0 | 0±0 | 0.62±0.16 | 73.91±97.84 |
| **SHISH BARAK** | 253.53±141.48 | 12.51±6.89 | 19.06±13.45 | 2.08±1.83 | 121.83±59.81 | 7.25±11.36 | 0.02±0.02 | 0.32±0.26 | 12.66±1.23 |
| **Madoos** | 6.64±0.67 | 0.33±0.03 | 0.66±0.07 | 0±0 | 3.9±0.39 | 0±0 | 0±0 | 0.29±0.08 | 1.73±0.42 |
| **HANEETH** | 0.35±0.03 | 0.08±0.1 | 0.04±0.01 | 0±0 | 0.63±1.04 | 0±0 | 0±0 | 0.13±0.13 | 0.04±0.04 |
| **JAREESH** | 41.38±42.71 | 9.68±11.89 | 0.79±0.09 | 9.13±11.79 | 6.29±1.05 | 1.65±2.59 | 0.02±0.03 | 0.12±0.06 | 0.61±0.02 |
| **TMMN** | 1515.1±1240.78 | 78.12±64.25 | 150.56±123.22 | 2.79±4.83 | 611.71±488.83 | 1.13±0.8 | 0.02±0.02 | 0.39±0.23 | 1.51±0.66 |
| **Raqsh** | 26.36±24.95 | 1.32±1.25 | 2.64±2.5 | 0±0 | 14.27±13.47 | 0.94±0.29 | 0.03±0.01 | 0.43±0.03 | 1.05±1.12 |
| **SALEEQ** | 63.8±31.19 | 12.61±3.46 | 2.56±2.49 | 11.33±2.5 | 14.36±13.56 | 2.32±0.7 | 0.04±0.02 | 0.17±0.06 | 0.87±0.15 |
| **Chicken Kabsah** | 154.16±36.36 | 13.12±5.8 | 13.19±2.12 | 6.52±4.88 | 73.39±12.09 | 1.55±1.26 | 0.03±0.03 | 0.37±0.1 | 1.78±0.07 |
| **MANDI** | 0.53±0.29 | 0.03±0.02 | 0.05±0.03 | 0±0 | 0.15±0.17 | 0.65±0.34 | 0.02±0.01 | 0.08±0.1 | 0.37±0.47 |
| **Threed** | 2087.34±744.81 | 104.36±37.24 | 208.73±74.48 | 0±0 | 874.61±319.13 | 0.67±0.23 | 0.02±0.01 | 0.47±0.11 | 1.75±0.49 |
| **MARQOQ** | 1263.49±317.28 | 63.17±15.87 | 126.35±31.72 | 0±0 | 605.89±108.88 | 0.37±0.06 | 0.01±0 | 0.45±0.2 | 3.5±1.01 |

Mineral composition of the 25 Saudi dish recipes based on ESHA software analysis. Supplemental Table 3:

|  | **Calcium (mg)** | **Chrom (mcg)** | **Copp (mg)** | **Fluor (mg)** | **Iodine (mcg)** | **Iron (mg)** | **Magnesium (mg)** | **Phos (mg)** | **Potassium (mg)** | **Zinc (mg)** |
| --- | --- | --- | --- | --- | --- | --- | --- | --- | --- | --- |
| **AREEKAH** | 16±2.59 | 0±0 | 0.2±0.05 | 0.02±0 | 14.27±13.07 | 2.25±0.18 | 47.68±14.93 | 139.68±33.66 | 167.66±43.35 | 1.24±0.35 |
| **SOUTHERN ASSIDAH** | 50.21±35.37 | 0.04±0.06 | 0.23±0.13 | 0.01±0 | 20.33±18.09 | 1.71±1.1 | 50.67±48.67 | 126.46±170.77 | 360.76±163.43 | 0.96±1.24 |
| **Saqu** | 3.55±0.32 | 0.1±0.01 | 0.02±0.01 | 0.04±0.01 | 0.56±0.06 | 0.17±0.02 | 1.96±0.37 | 5.95±1.43 | 17.46±2.8 | 0.03±0.01 |
| **Jamriah** | 159.94±84.5 | 0.03±0.02 | 0.14±0.06 | 0.03±0.01 | 18.93±7.01 | 2.58±0.81 | 43.44±8.61 | 177±38.08 | 119.83±51.93 | 0.81±0.34 |
| **MANSAF** | 10.75±3.71 | 0.01±0.01 | 0.06±0.02 | 0±0 | 6.47±5.83 | 1.05±0.2 | 13.89±1.58 | 81.74±27.31 | 130.56±43.27 | 1.43±0.66 |
| **MARASYAA** | 136.94±135.09 | 0.03±0.01 | 0.14±0.04 | 0±0 | 74.2±25.99 | 3.36±1.88 | 37.02±15.02 | 159.52±82.37 | 122.8±34.47 | 0.84±0.26 |
| **HANINI** | 66.38±85.63 | 0.06±0.03 | 0.13±0.07 | 0±0 | 0.16±0.02 | 0.7±0.82 | 21.06±14.19 | 50.92±59.88 | 216.16±108.71 | 0.25±0.24 |
| **MASOOB** | 10.72±7.06 | 0.37±0.06 | 0.14±0.09 | 0.01±0.01 | 4.57±4.71 | 0.96±0.73 | 38.27±22.78 | 80.44±62.89 | 266.14±54.67 | 0.73±0.58 |
| **SAYADIAH** | 21.86±7.37 | 0.07±0.07 | 0.08±0.05 | 0±0 | 21.88±11.61 | 1±0.77 | 25.24±18.54 | 131.1±87.13 | 173.25±95.48 | 0.54±0.44 |
| **MANTU** | 17.02±5.61 | 0.05±0.06 | 0.07±0.03 | 0.01±0.02 | 7.33±11.04 | 1.61±0.61 | 14.4±4.82 | 69.61±30.05 | 135.07±61.05 | 0.31±0.06 |
| **MUTABBAQ** | 55±36.94 | 0.2±0.08 | 0.07±0.01 | 0±0.01 | 28.14±4.92 | 1.33±0.27 | 14.1±1.51 | 69.37±10.08 | 145.84±22.02 | 0.52±0.16 |
| **Meat Kabsah** | 8.01±0.46 | 0.08±0.08 | 0.02±0 | 0.03±0.01 | 10.74±8.81 | 0.75±0.14 | 9.21±1.02 | 58.12±6.08 | 145.21±22.37 | 0.98±0.11 |
| **HASAWI** | 12.53±1.35 | 0.09±0.03 | 0.03±0.02 | 0±0 | 30.93±14.07 | 0.66±0.17 | 11.61±4.21 | 53.52±13.57 | 158.18±31.36 | 0.79±0.16 |
| **KBEBAH HAIL** | 46.91±6.87 | 0.08±0.04 | 0.08±0.01 | 0±0 | 13.87±7.38 | 0.93±0.29 | 17.92±2.5 | 34.39±19.11 | 143.18±10.78 | 0.38±0.28 |
| **SHISH BARAK** | 64.63±10.14 | 0.02±0.01 | 0.04±0.02 | 0.02±0.02 | 22.94±4.39 | 0.91±0.46 | 7.09±2.7 | 38.18±17.3 | 63.51±25.71 | 0.14±0.07 |
| **Madoos** | 10.2±0.72 | 0.02±0.01 | 0.11±0.02 | 0.04±0.01 | 18.77±6.14 | 1.09±0.18 | 8.04±1.29 | 38.27±9.68 | 101.19±22.56 | 0.44±0.11 |
| **HANEETH** | 7.98±0.58 | 0.01±0 | 0.15±0.01 | 0±0 | 31.83±10.27 | 1.77±0.07 | 0.15±0.15 | 103.43±5.7 | 222.58±13.19 | 2.29±0.12 |
| **JAREESH** | 10.35±4.24 | 0.05±0.03 | 0.02±0.01 | 0±0 | 21.8±12.61 | 0.32±0.09 | 5.63±1.41 | 30.57±11.33 | 65.87±13.78 | 0.24±0.14 |
| **TMMN** | 12.37±5.25 | 0.05±0.01 | 0.03±0.01 | 0±0 | 19.49±6.82 | 0.64±0.1 | 12.46±2.94 | 49.32±6.41 | 165.14±20.74 | 0.65±0.29 |
| **Raqsh** | 9.77±1.05 | 0.03±0.02 | 0.05±0.02 | 0.03±0.01 | 9.43±3.78 | 0.94±0.09 | 16.63±1.79 | 83.38±11.86 | 147.91±20.14 | 1.27±0.26 |
| **SALEEQ** | 15.61±2.15 | 0.02±0.01 | 0.03±0.01 | 0.04±0.01 | 5.73±6.24 | 0.54±0.05 | 8.19±1.65 | 50.53±10.66 | 68.52±22.07 | 0.4±0.1 |
| **Chicken Kabsah** | 7.99±0.47 | 0.07±0.02 | 0.04±0.01 | 0.03±0.01 | 6.61±5.16 | 0.37±0.14 | 7.79±0.77 | 36.99±6.94 | 108.75±8.1 | 0.28±0.13 |
| **MANDI** | 4.18±0.81 | 0±0.01 | 0.01±0 | 0.04±0.01 | 17.7±1.85 | 0.54±0.18 | 5.51±2.38 | 40.27±20.97 | 69.68±36.67 | 0.72±0.37 |
| **Threed** | 15.98±2.15 | 0.04±0.01 | 0.04±0.01 | 0.02±0 | 4.84±0.97 | 0.69±0.07 | 14.85±0.46 | 57.02±10.77 | 200.23±2.26 | 0.84±0.23 |
| **MARQOQ** | 13.12±0.97 | 0.1±0.05 | 0.07±0.01 | 0.03±0.01 | 22.72±5.5 | 0.86±0.1 | 19.83±2.14 | 65.66±2.92 | 195.81±40.9 | 0.77±0.02 |
