# Supplementary material for: Comprehensive nutritional compositions of traditional dishes from five Saudi regions using Elizabeth Stewart Hands and Associates Food Processor software
Source: Front Nutr. 2025 Nov 10;12:1658078. doi: 10.3389/fnut.2025.1658078 (PMC12641437; doi:10.3389/fnut.2025.1658078)
Supplement: Supplementary file 2 [file Data_Sheet_1.PDF]

Food

Amounts

Booklet

Interviewer Copy

Food Amounts Booklet
Conversion Guide for Dietary Interviewers

When the participant shows a picture in the Food Amounts Booklet, convert to NDSR amounts per the following information.

Squares and Rectangles 1/4 inch grid (pg 1)	Circles (pg 2)		
Enter as fractions, e.g., 9/4 wide x 12/4 long x 5/4 high or use page 4 for thickness.<br>Each square is 1/4" x 1/4".<br>Select shape, cube (3 dimensions) or rectangle (2 dimensions) per NDSR.	Select shape, circle or sphere.<br>Enter diameter in inches (") per the following:		
	A = 1 inch<br>B = 2 inches<br>C = 2 1/2 inches	D = 3 inches<br>E = 4 inches<br>F = 5 inches	G = 6 inches<br>H = 7 inches

Wedges (pg 3)						
**STEP 1: Select the number that corresponds to the radius. Enter the radius in inches in NDSR**	**STEP 2: Find the number selected in step 1. Next, find the letter that matches to the length of the arc. Enter the number in inches you obtained as the width in NDSR**					
		**A**	**B**	**C**	**D**	**E**
**1** = 4" radius (8" D[1])	**1**	0.6"	1.1"	2.1"	2.6"	3.9"
**2** = 4 1/2" radius (9" D)	**2**	0.7"	1.3"	2.4"	2.9"	4.4"
**3** = 6" radius (12" D)	**3**	0.9"	1.6"	3.1"	3.9"	5.9"
**4** = 8" radius (16" D)	**4**	1.1"	2.1"	4.1"	5.1"	7.8"
**5** = 9" radius (18" D)	**5**	1.3"	2.4"	4.7"	5.8"	8.9"
For 3D[2] wedge, also use **Thickness** (pg 4, height=x/16) or **Squares and Rectangles** (pg 1, height=x/4)						

Thickness (pg 4)	Measuring Spoons (pg 5)	Eating and Serving Spoons (pg 6)	Measuring Cups (pg 7)
Each unit is 1/16" thick.<br>Enter thickness as fraction.<br>E.g.: 1 = 1/16"<br>2 = 2/16"<br>3 = 3/16"<br>18 = 18/16"	Standard measures<br>1/2 teaspoon (TS)<br>1 teaspoon<br>1/2 tablespoon (TB)<br>1 tablespoon	Teaspoons:<br>Level = 1 TS<br>Heaping = 2 TS<br>Tablespoon:<br>Level = 1 TB<br>Heaping = 2 TB	Standard measures<br>1/4 cup (CP)<br>1/3 CP<br>1/2 CP<br>1 CP

Glasses (pg 8-9) (Fluid Ounces, FO)			
1 A = 1 1/4 FO<br>1 B = 2 1/2 FO<br>1 C = 3 3/4 FO<br>1 D = 5 FO	2 A = 2 1/2 FO<br>2 B = 5 FO<br>2 C = 7 1/2 FO<br>2 D = 10 FO	3 A = 3 FO<br>3 B = 6 FO<br>3 C = 9 FO<br>3 D = 12 FO	4 A = 8 FO (1 CP)<br>4 B = 16 FO (2 CP)<br>4 C = 24 FO (3 CP)<br>4 D = 32 FO (4 CP)

Bowls (pg 10-11)				Wine Glass (pg12)	Mug (pg 13)
1 A = 1/8 CP<br>1 B = 1/4 CP<br>1 C = 3/8 CP<br>1 D = 1/2 CP	2 A = 3/8 CP<br>2 B = 3/4 CP<br>2 C = 1 1/4 CP<br>2 D = 1 1/2 CP	3 A = 3/4 CP<br>3 B = 1 1/2 CP<br>3 C = 2 1/4 CP<br>3 D = 3 CP	4 A = 2 CP<br>4 B = 4 CP<br>4 C = 6 CP<br>4 D = 8 CP	A = 2 FO<br>B = 4 FO<br>C = 6 FO<br>D = 8 FO	A = 2 1/2 FO<br>B = 5 FO<br>C = 7 1/2 FO<br>D = 11 FO

Mounds (pg 14-15)	Meats (pg 16) (Ounces, OZ)	Chicken (pg 17) (Medium, MD)	Fish (pg 18)
1 = 1 CP<br>2 = 3/4 CP<br>3 = 1/2 CP<br>4 = 1/3 CP<br>5 = 1/4 CP	All are edible portion<br>1 = 3 OZ<br>2 = 3 OZ<br>3 = 1 1/2 OZ	Edible portion<br>Wing = 1 MD<br>Drumstick = 1 MD<br>Thigh = 1 MD<br>Breast = 1 MD	Edible portion<br>1 = 2 OZ<br>2 = 3 OZ<br>3 = 1/2 OZ<br>4 = 1 OZ

[1] D = Diameter
[2] 3D = three dimensional

Squares and Rectangles

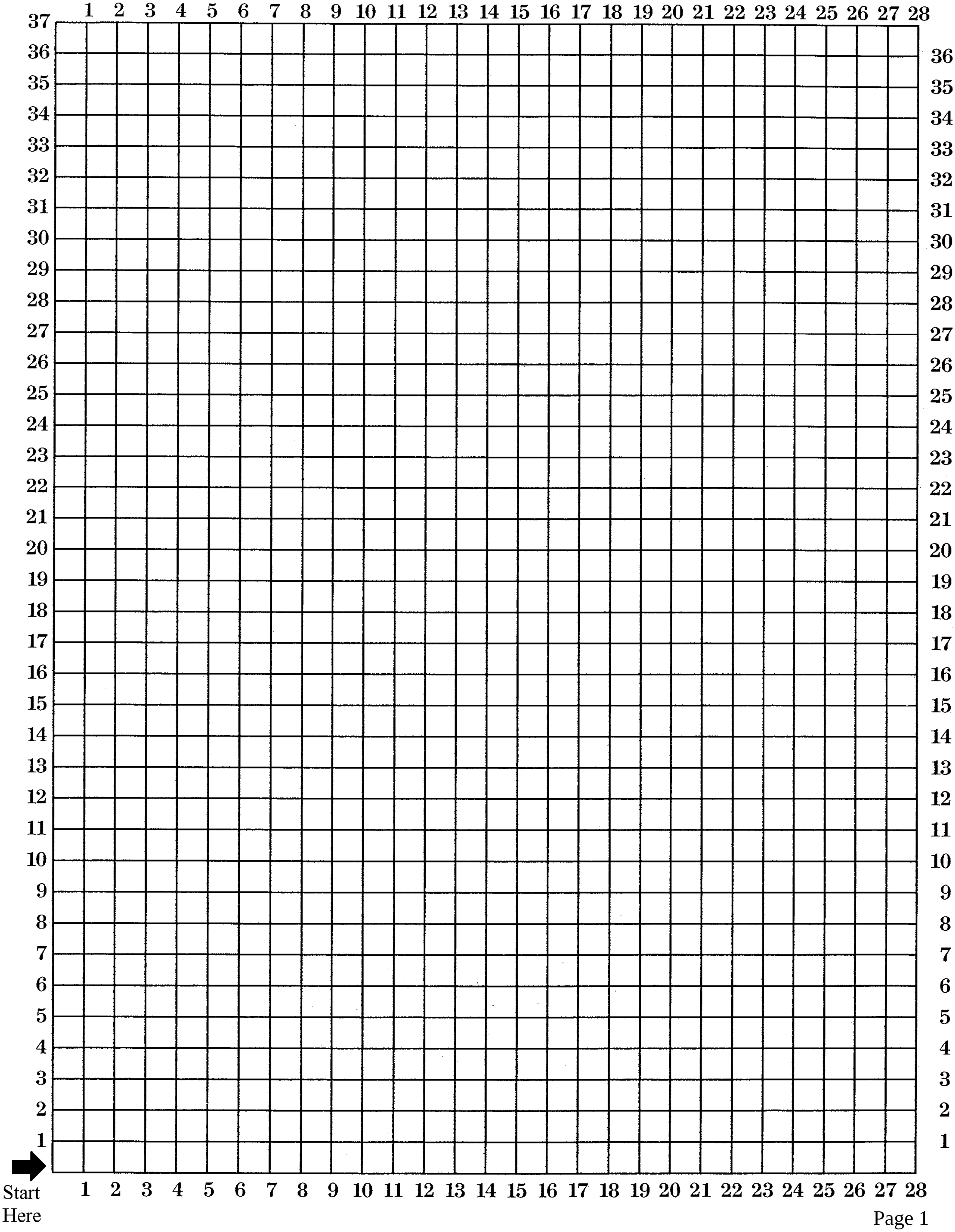

Circles

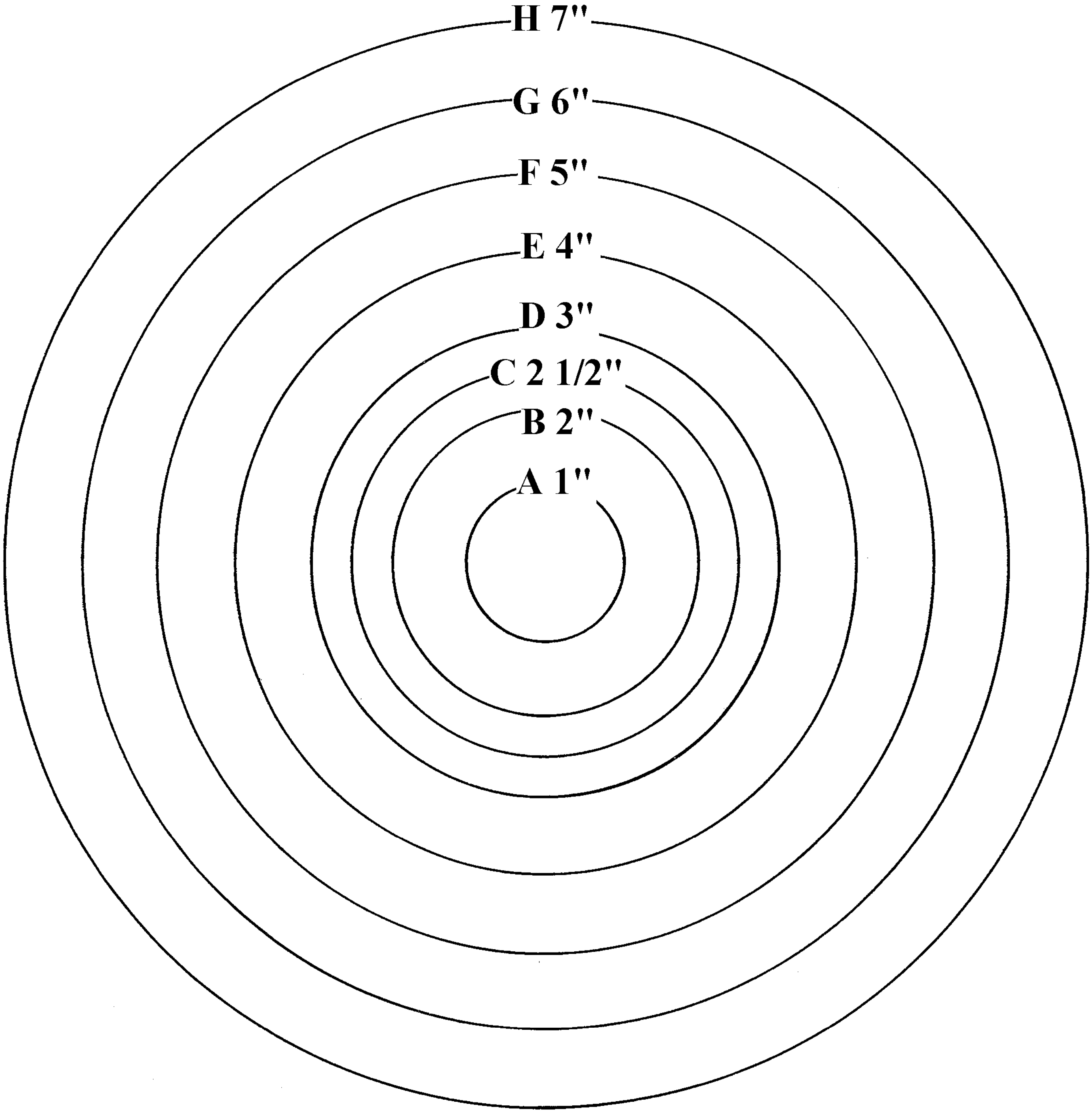

Wedges

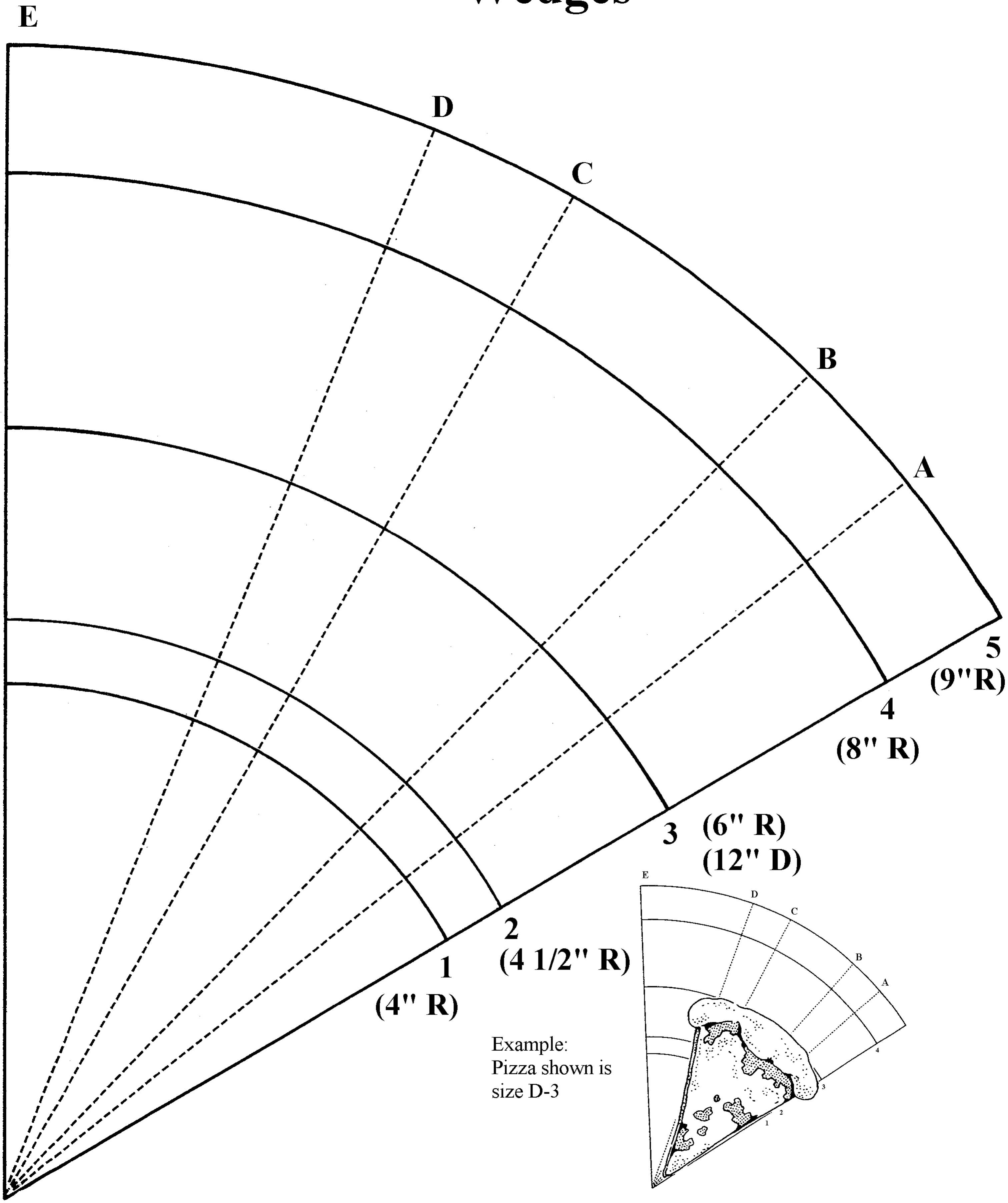

Example:
Pizza shown is
size D-3

Thickness

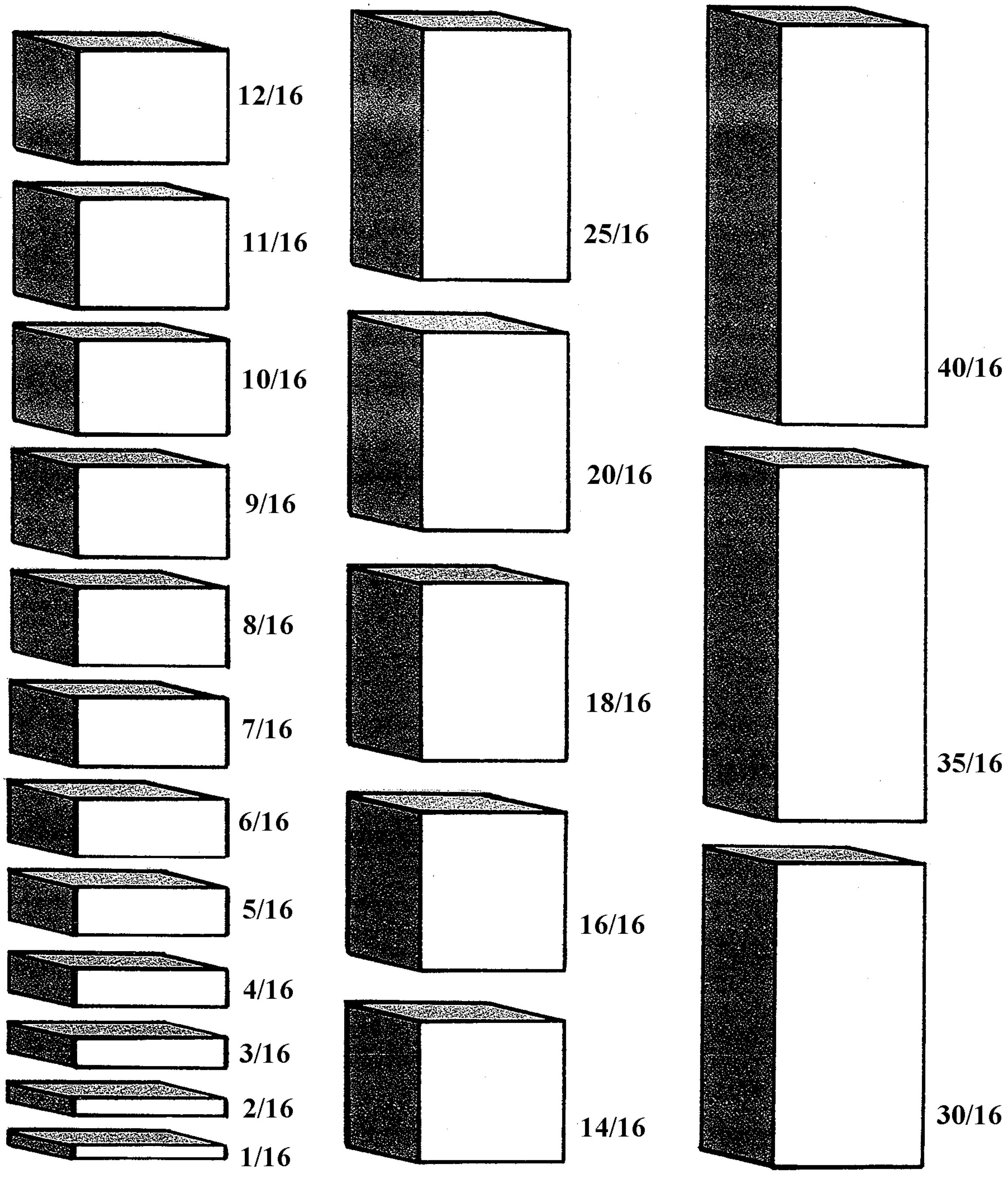
12/16
11/16
10/16
9/16
8/16
7/16
6/16
5/16
4/16
3/16
2/16
1/16
25/16
20/16
18/16
16/16
14/16
40/16
35/16
30/16

Measuring Spoons

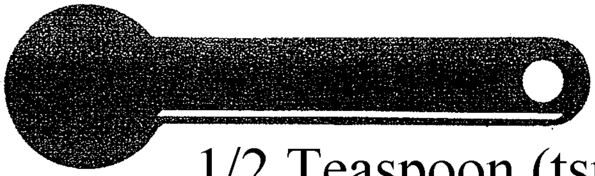

1/2 Teaspoon (tsp)

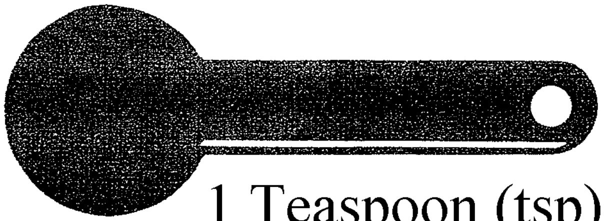

1 Teaspoon (tsp)

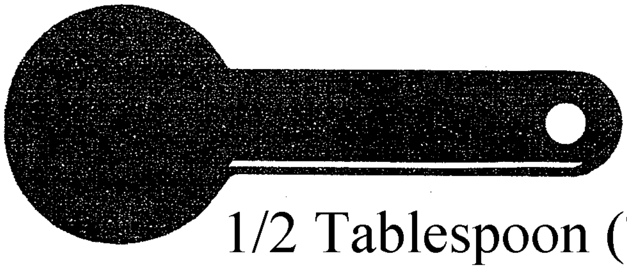

1/2 Tablespoon (Tbsp)

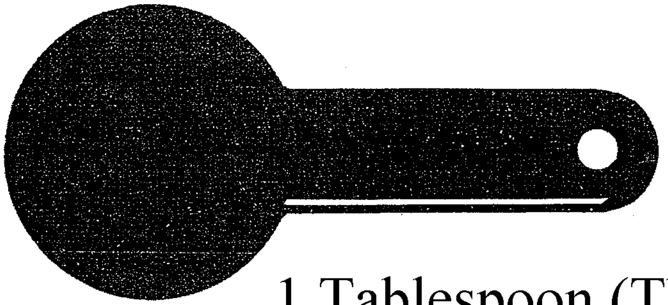

1 Tablespoon (Tbsp)

Eating and Serving Spoons

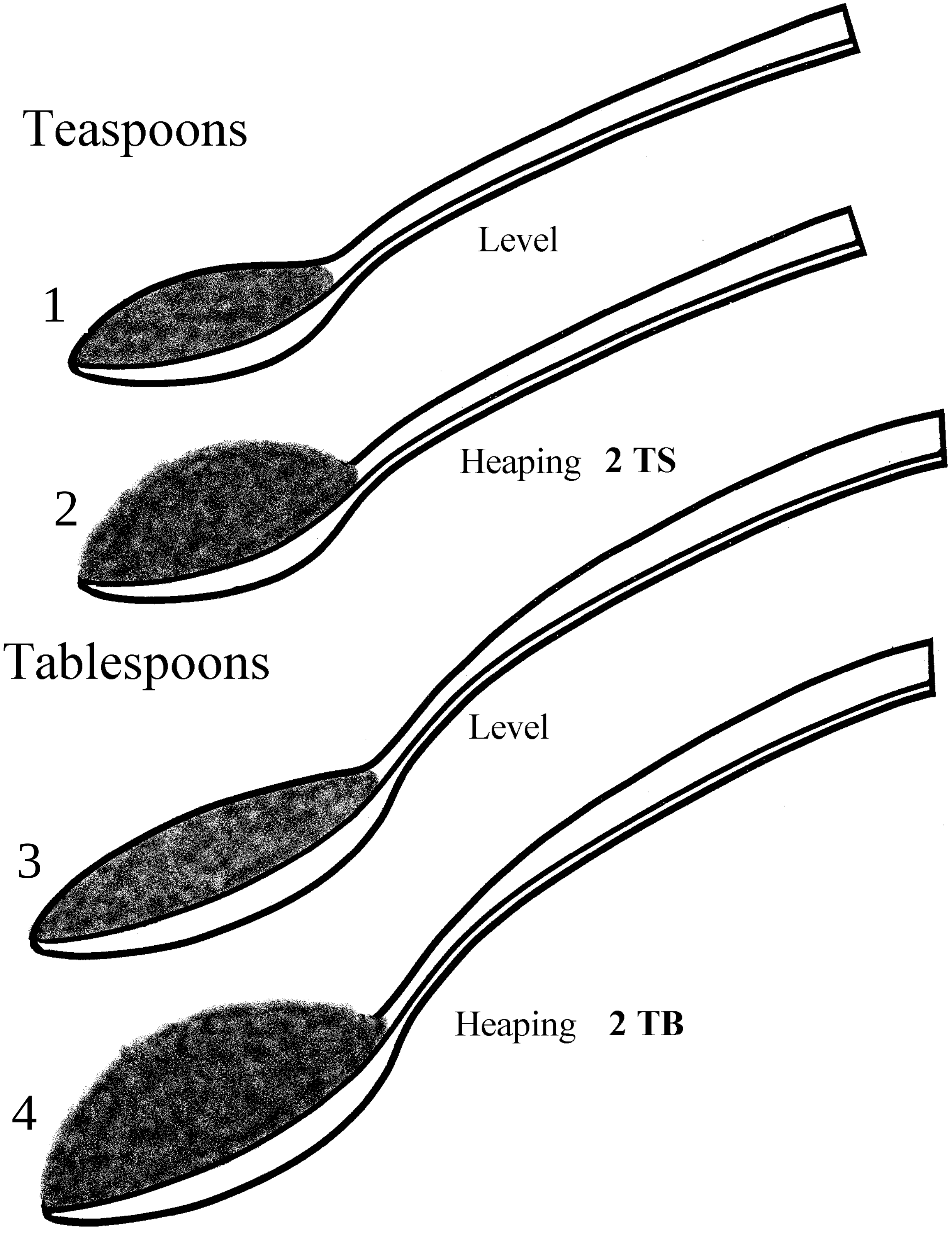

Measuring Cups

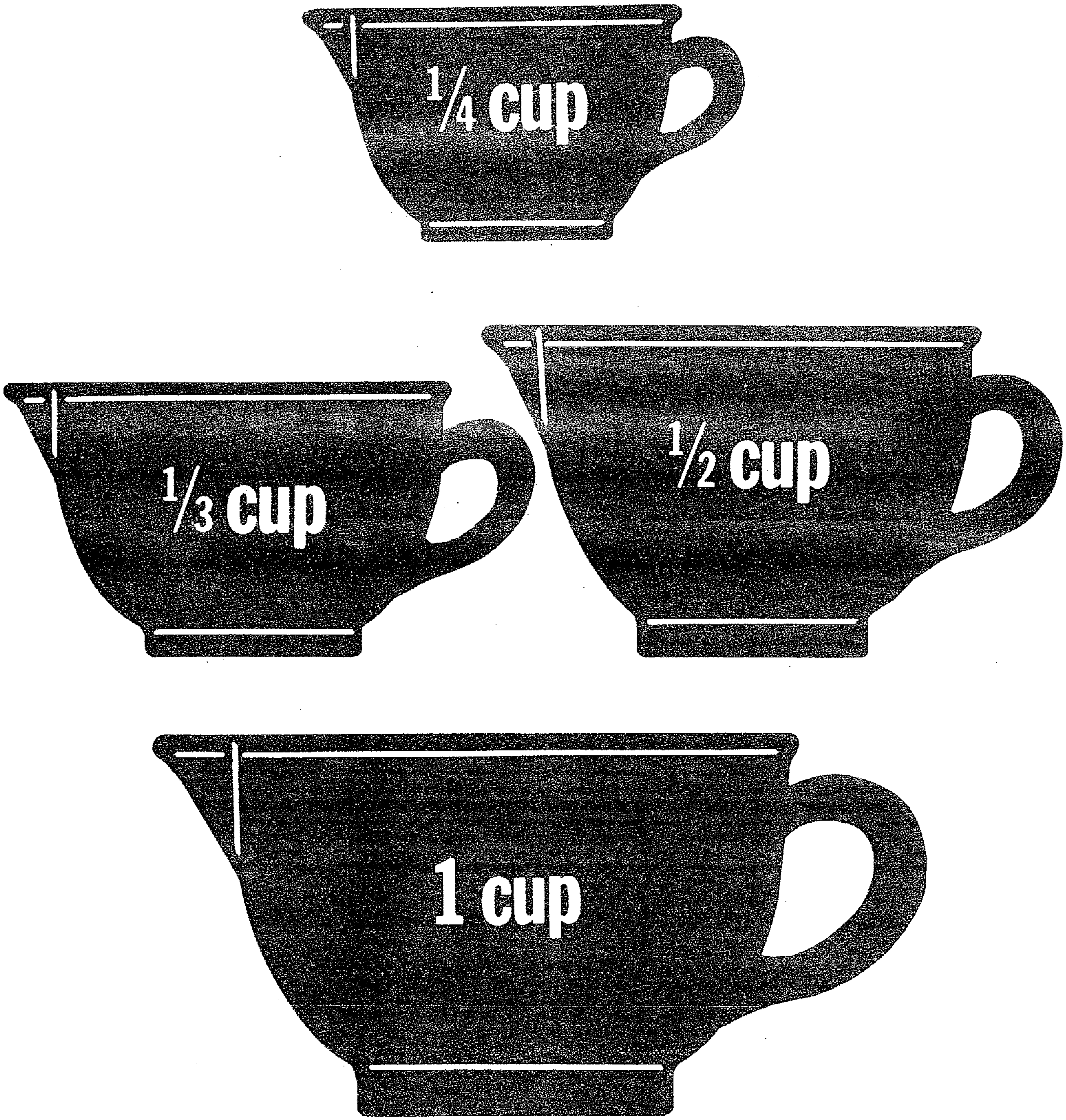

Glasses

1

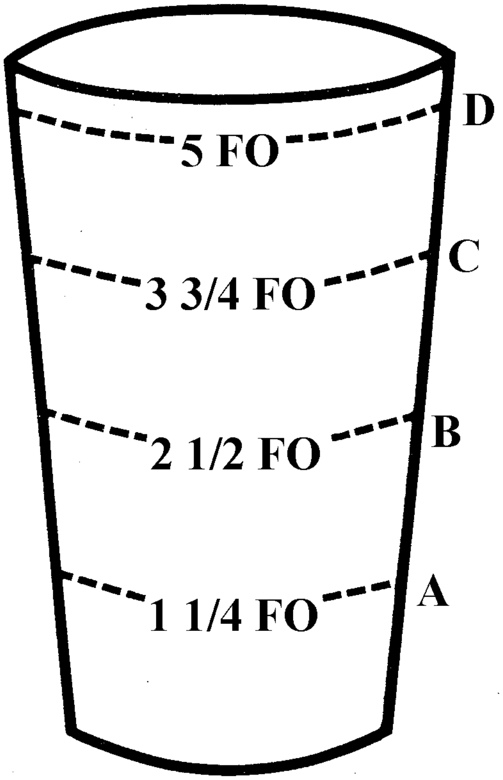
D
5 FO
C
3 3/4 FO
B
2 1/2 FO
A
1 1/4 FO

3

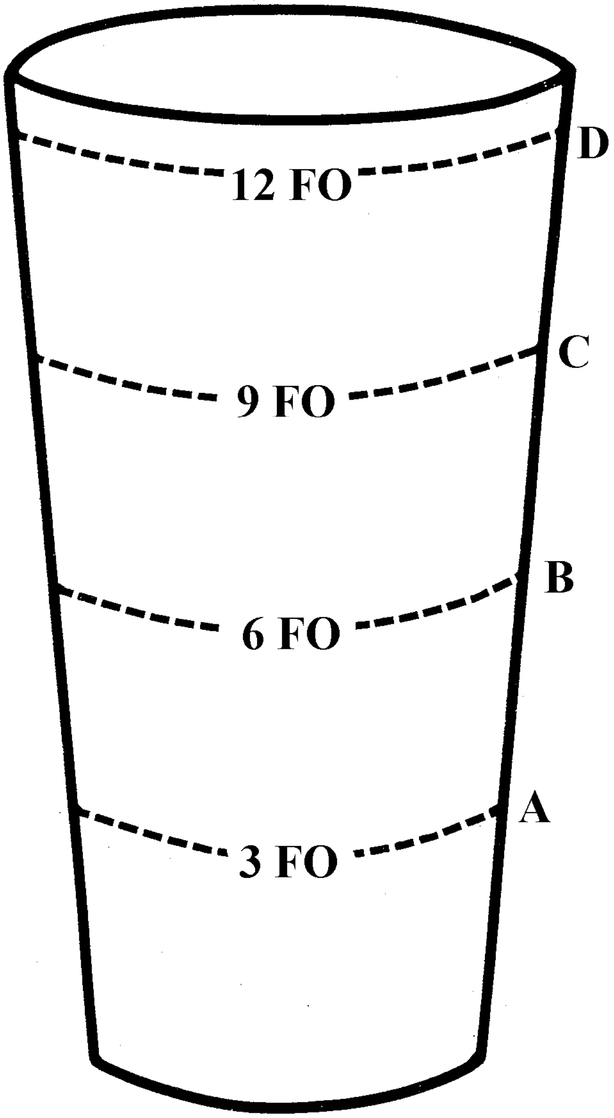
D
12 FO
C
9 FO
B
6 FO
A
3 FO

2

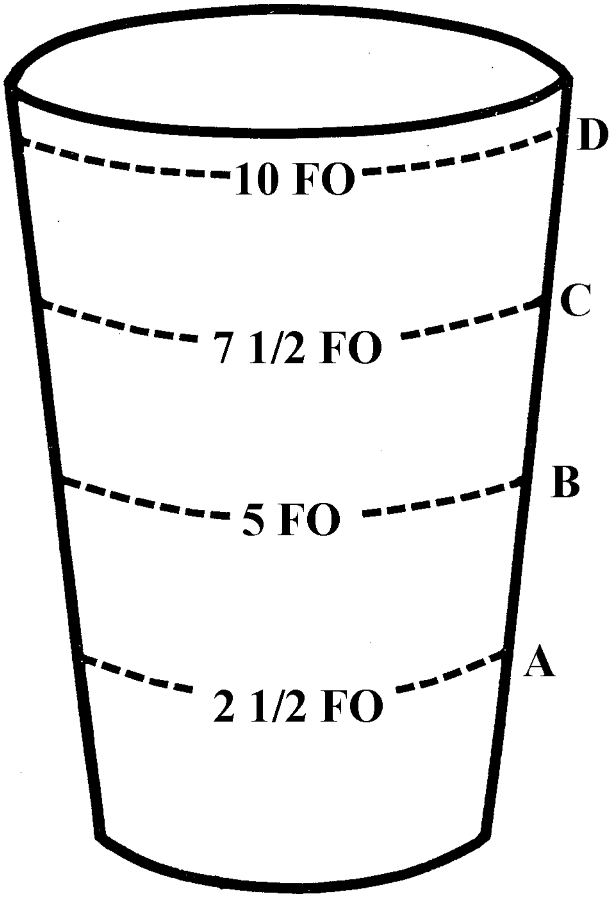
D
10 FO
C
7 1/2 FO
B
5 FO
A
2 1/2 FO

Glasses

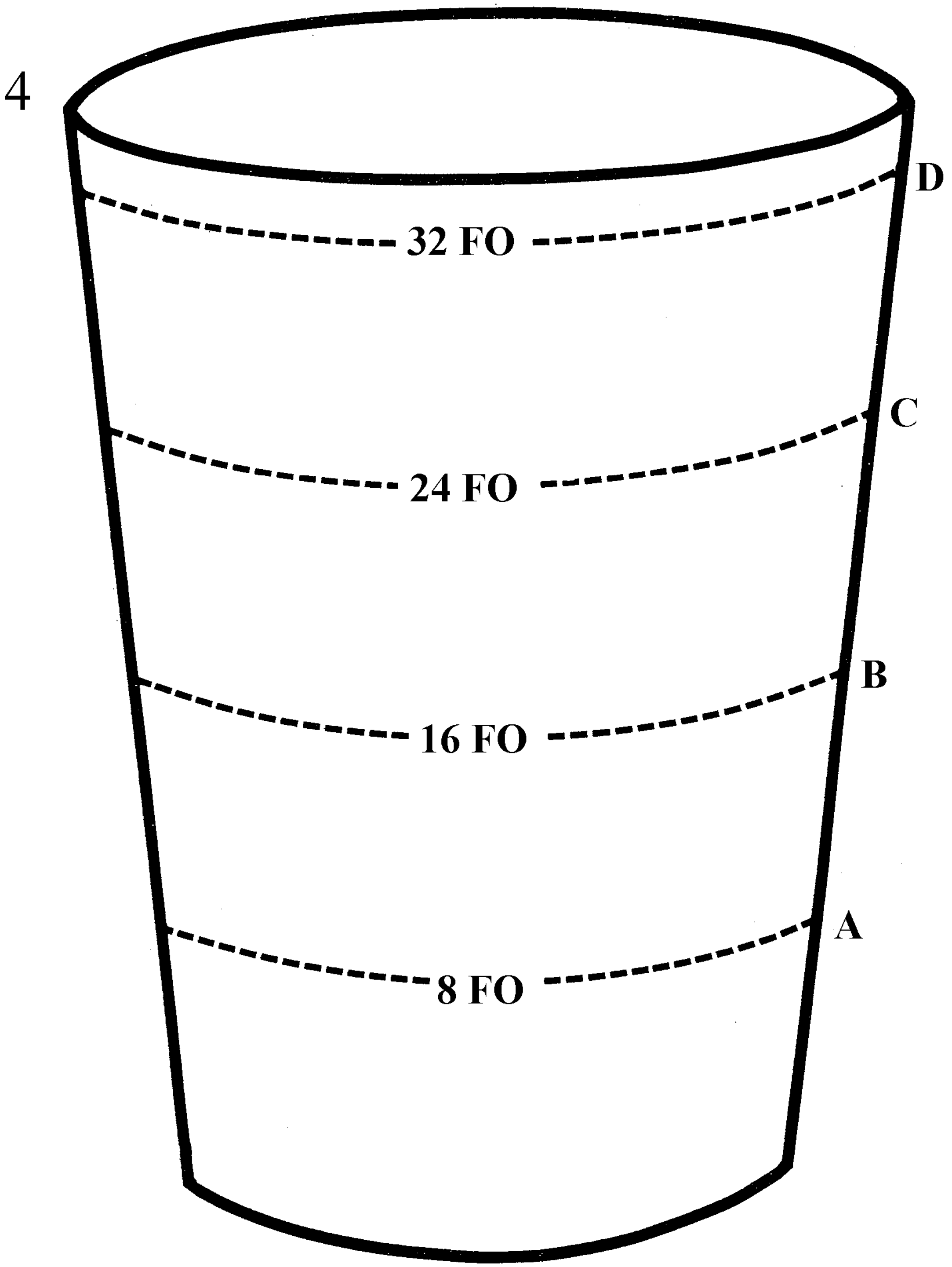

Bowls

1

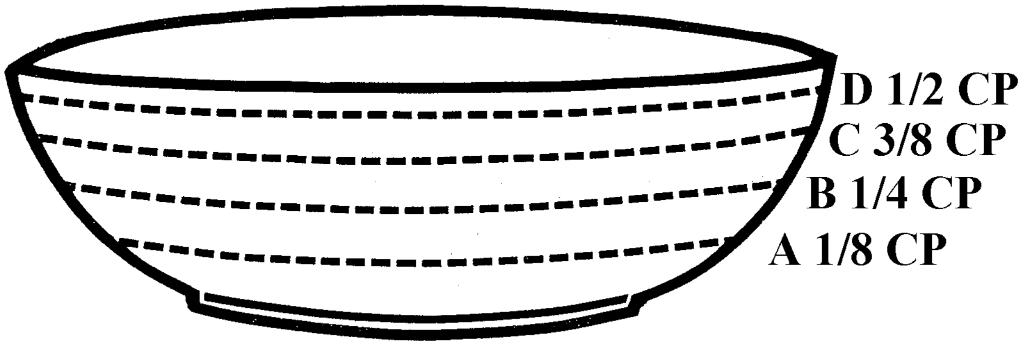
D 1/2 CP
C 3/8 CP
B 1/4 CP
A 1/8 CP

2

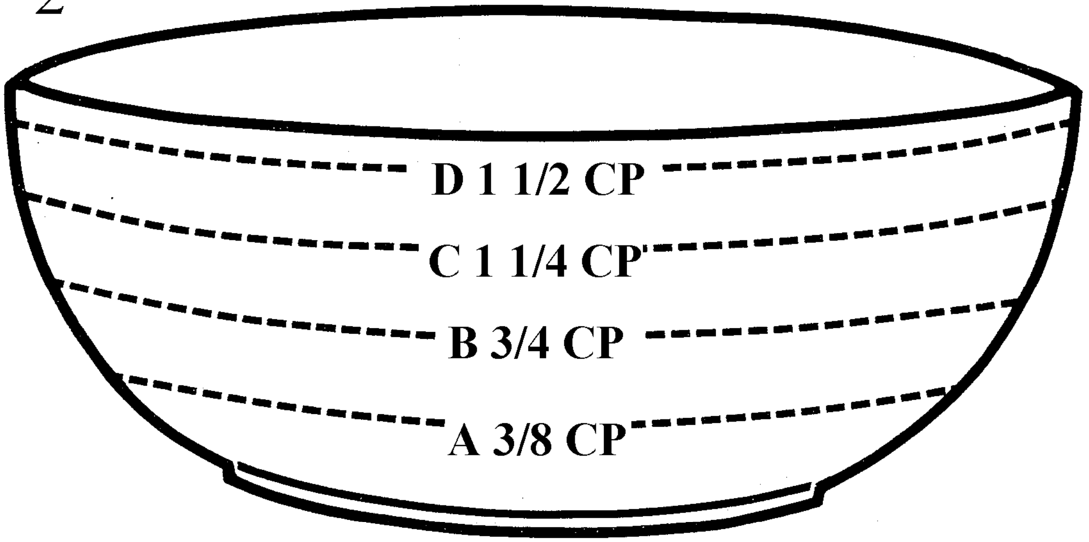
D 1 1/2 CP
C 1 1/4 CP
B 3/4 CP
A 3/8 CP

3

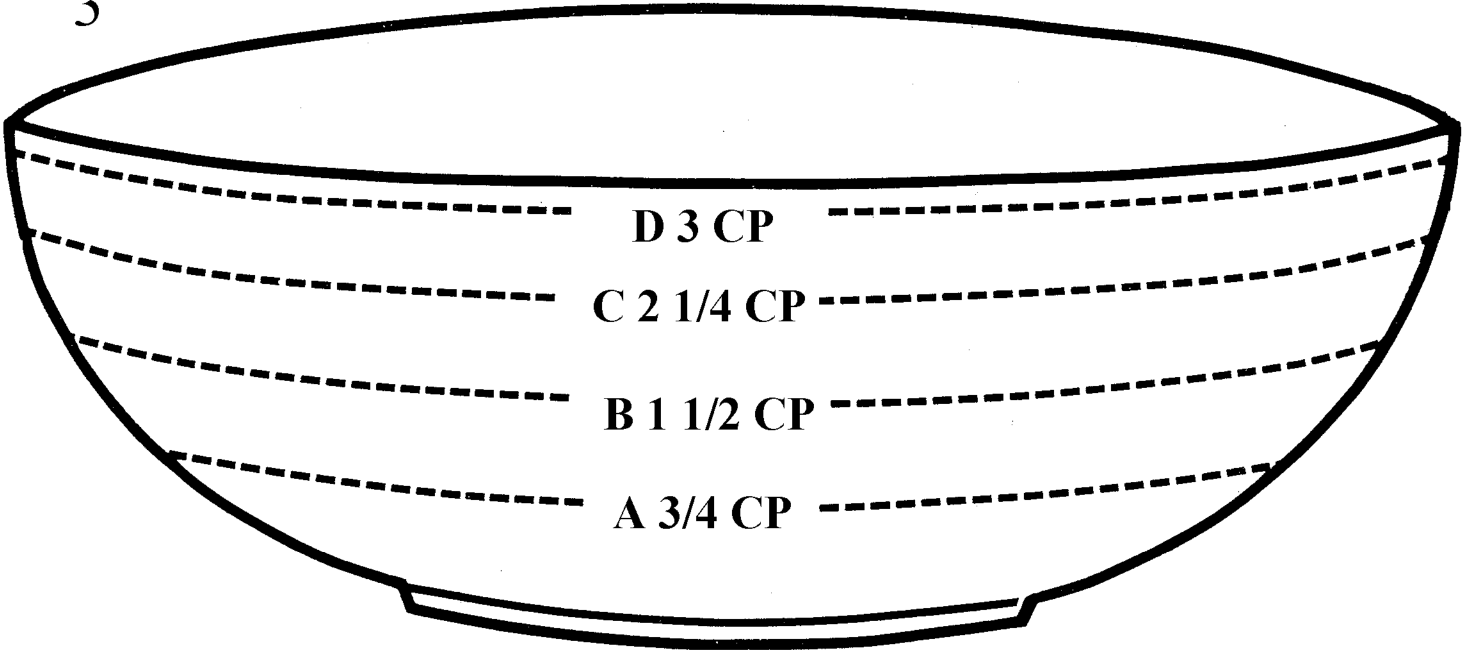
D 3 CP
C 2 1/4 CP
B 1 1/2 CP
A 3/4 CP

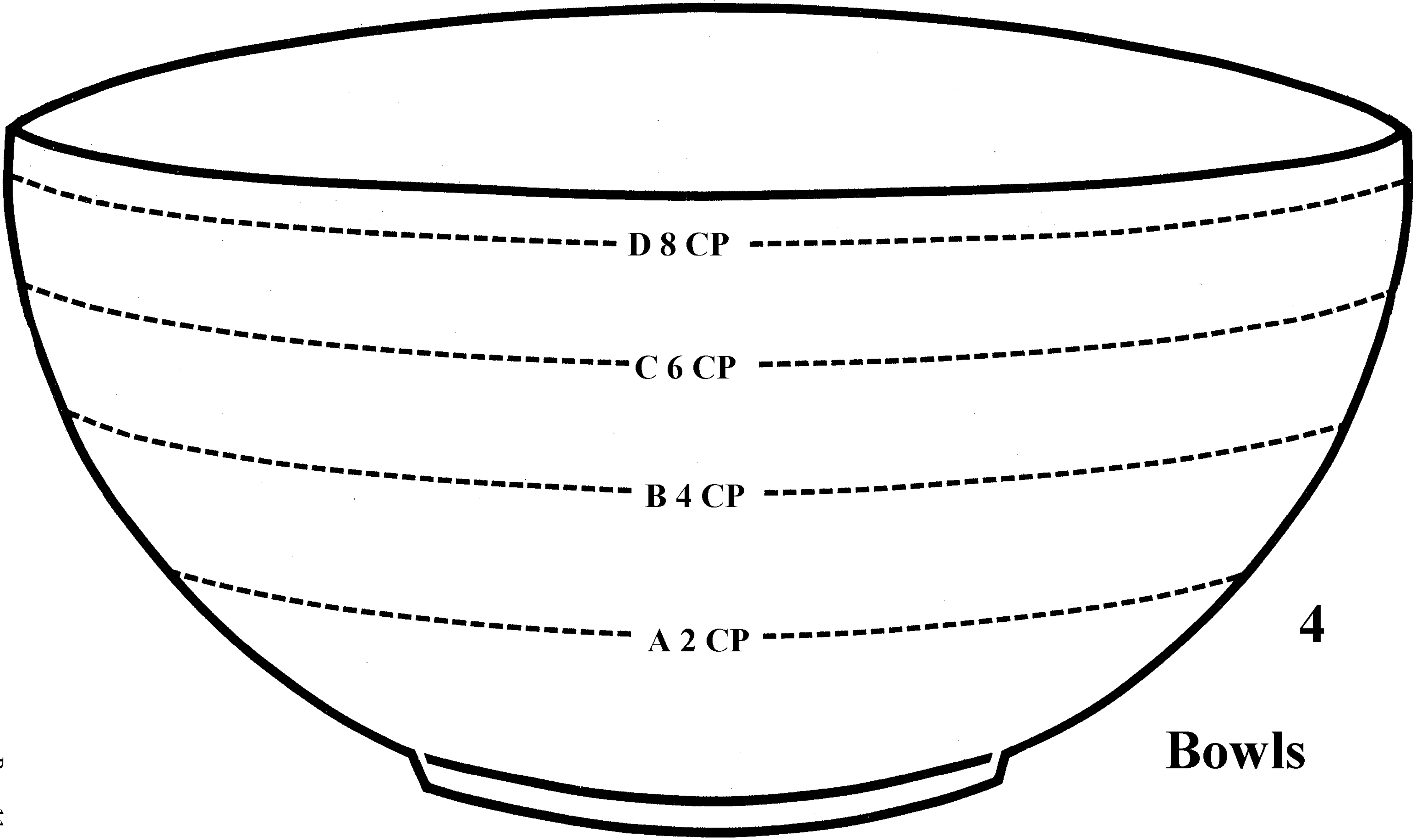

4

Bowls

Wine Glass

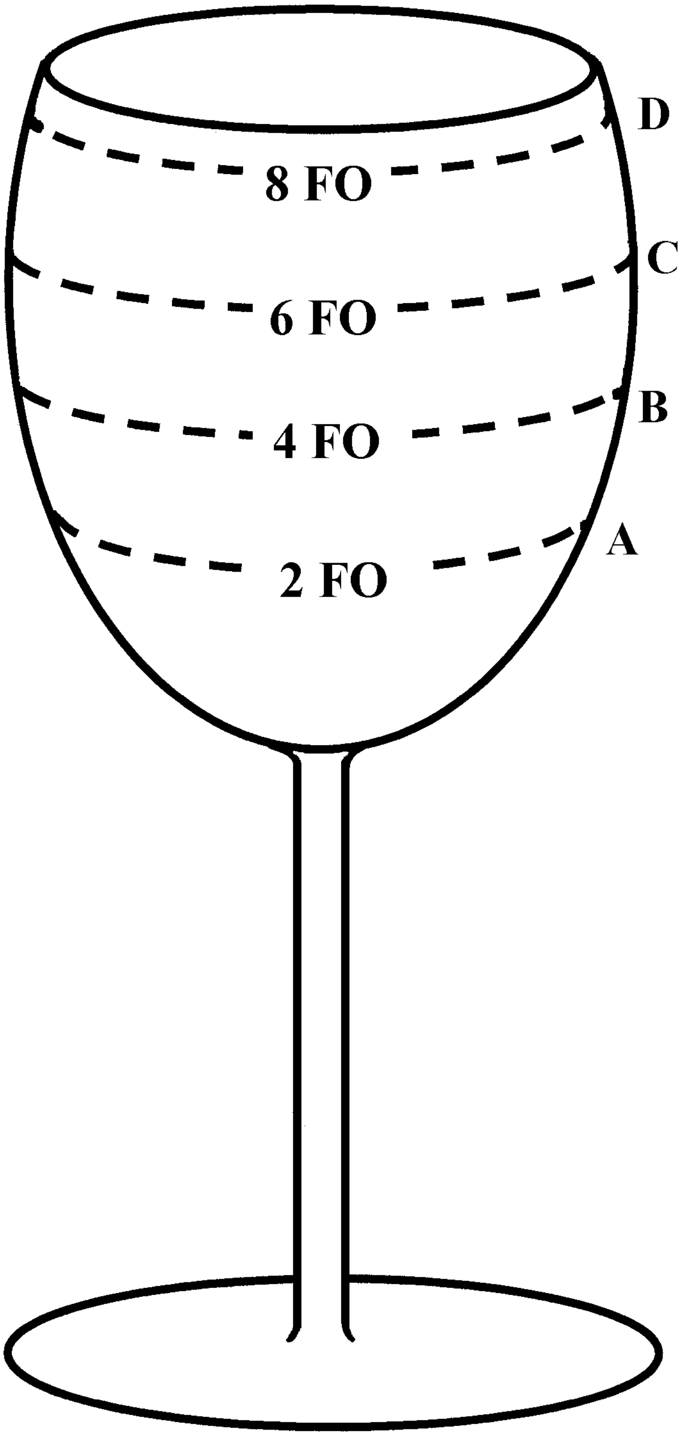

Mug

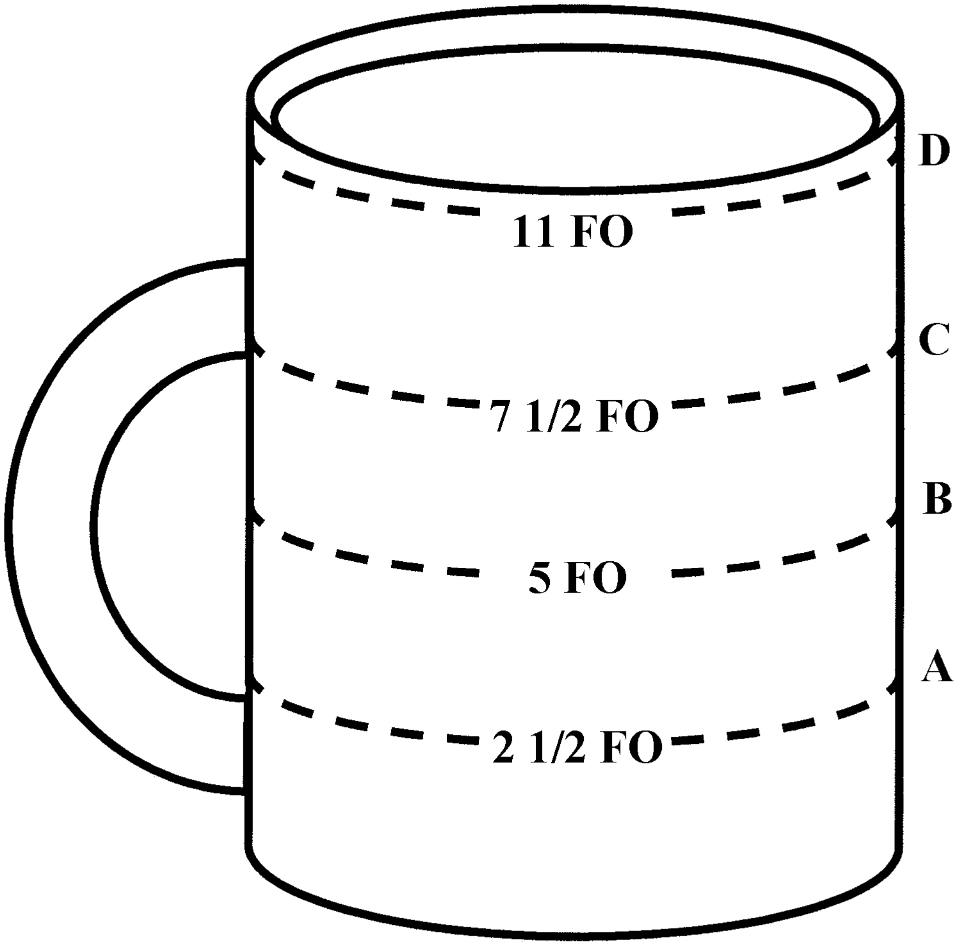

Mounds

1

1 CP

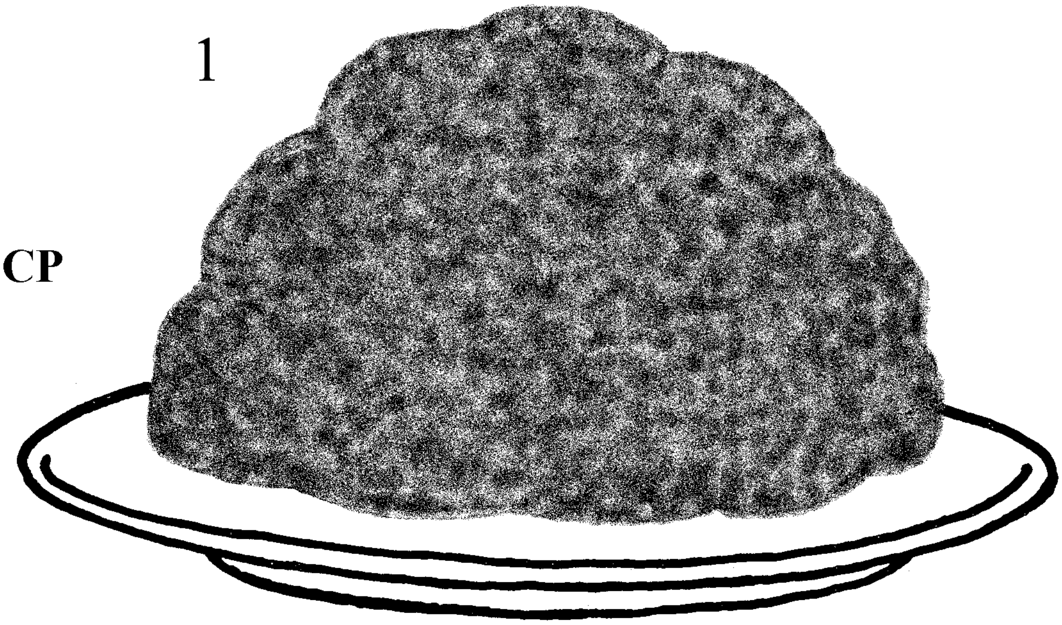

2

3/4 CP

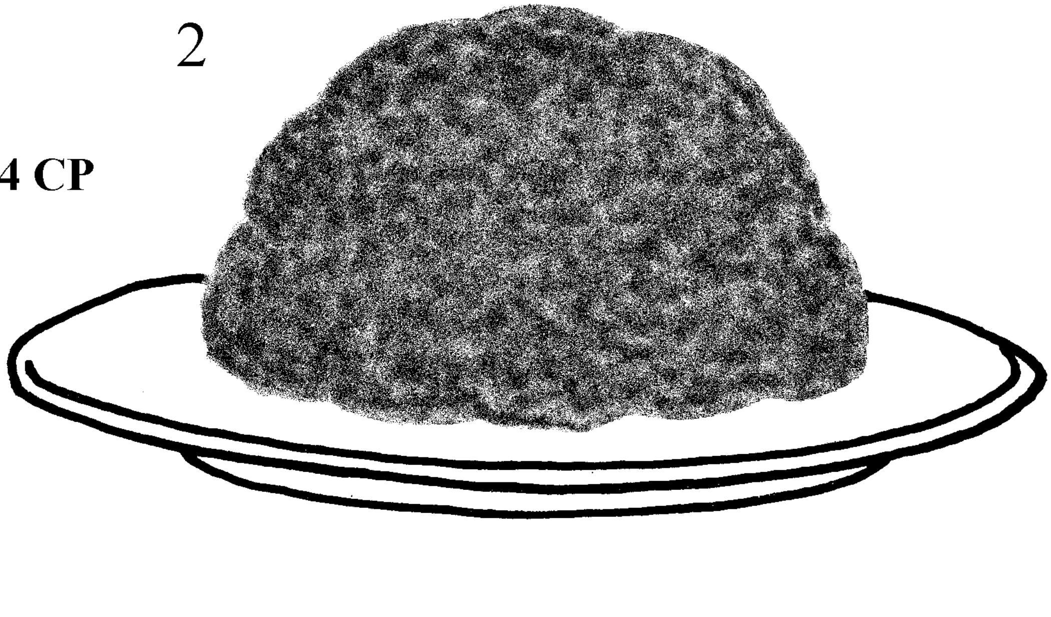

Mounds

3

1/2 CP

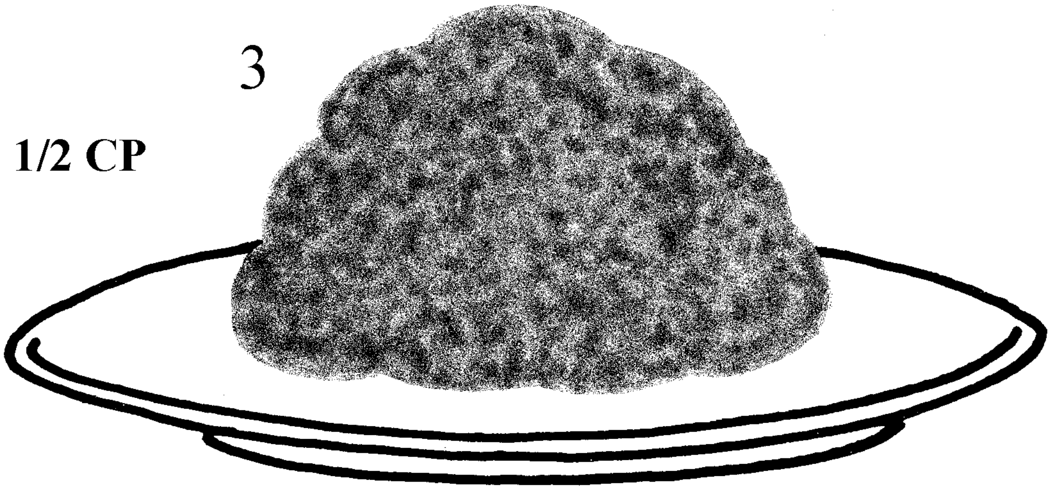

4

1/3 CP

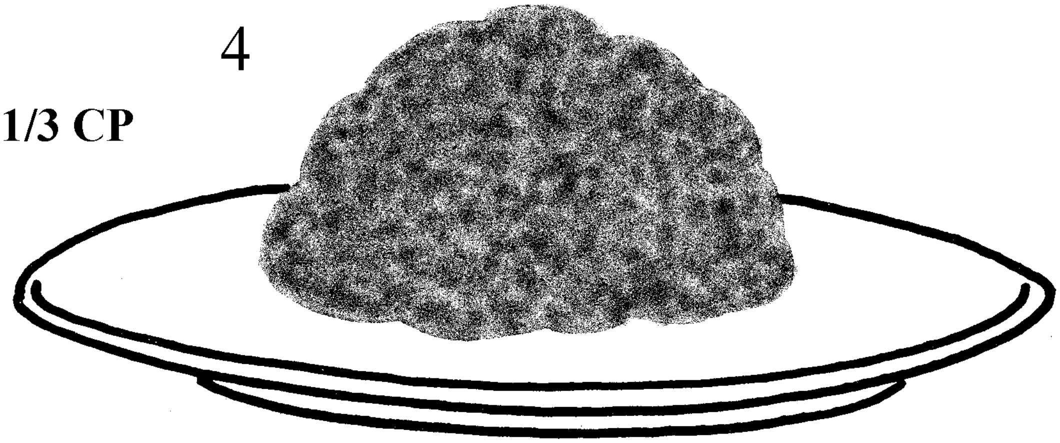

5

1/4 CP

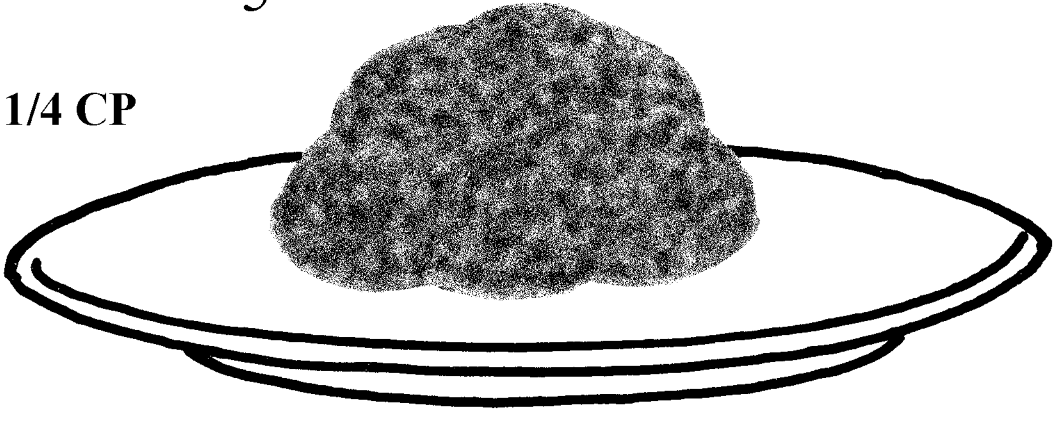

Meats

1

3 OZ

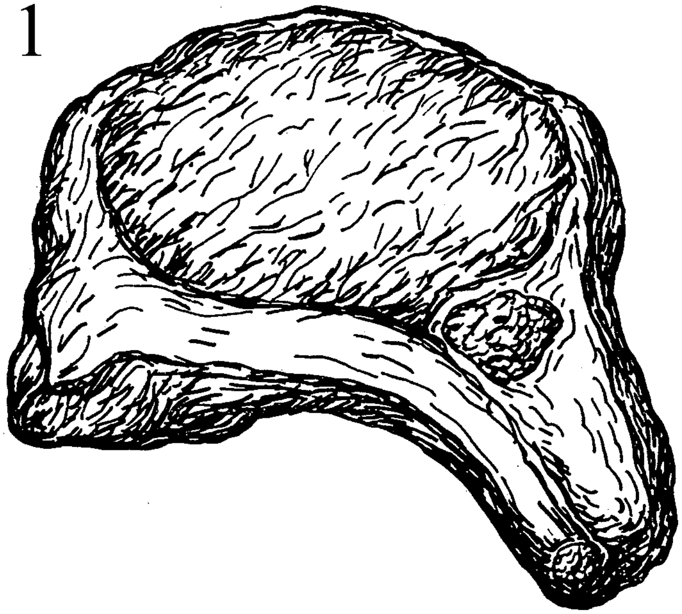

2

3 OZ

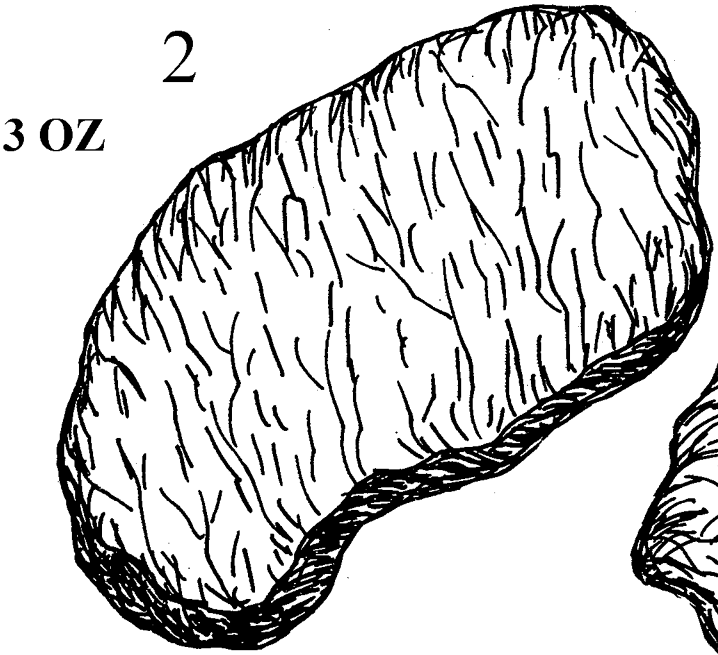

1 1/2 OZ

3

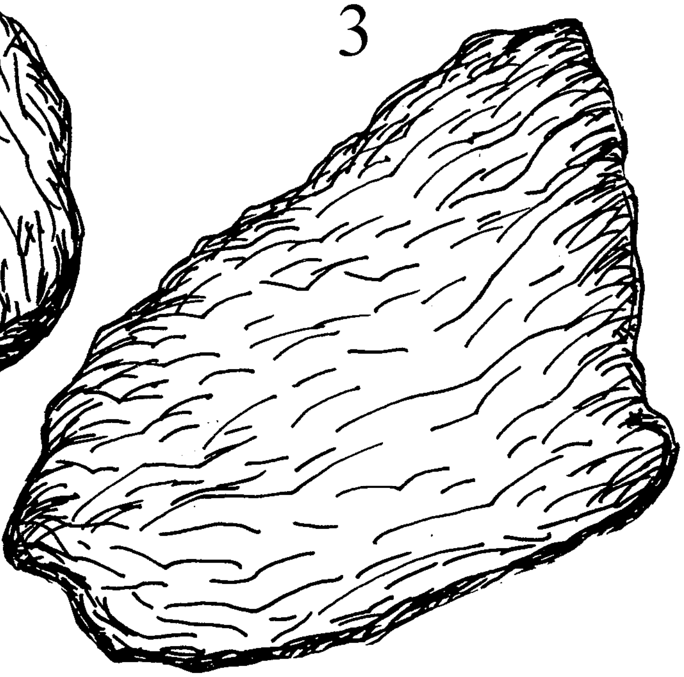

Chicken

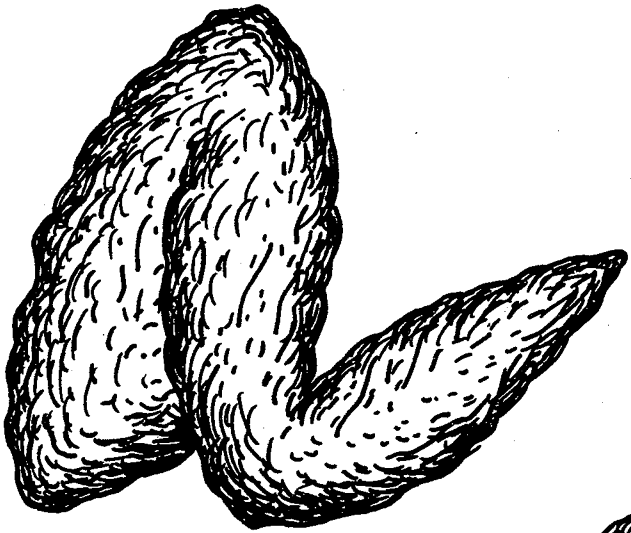

1 Wing

MD

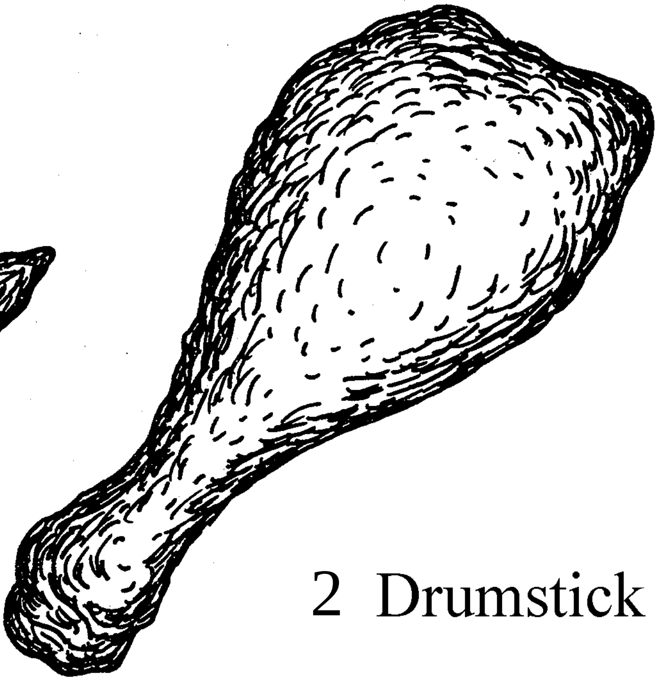

2 Drumstick

MD

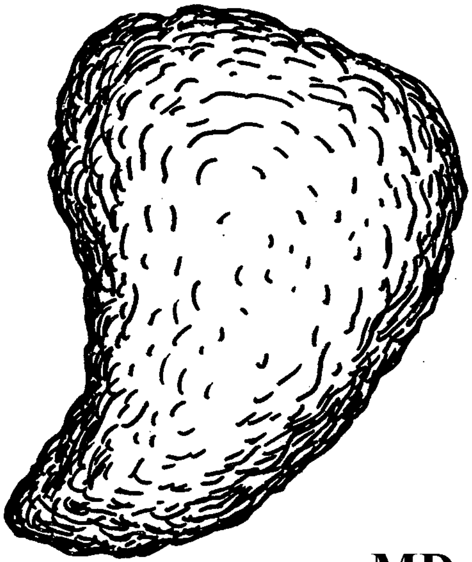

MD

3 Thigh

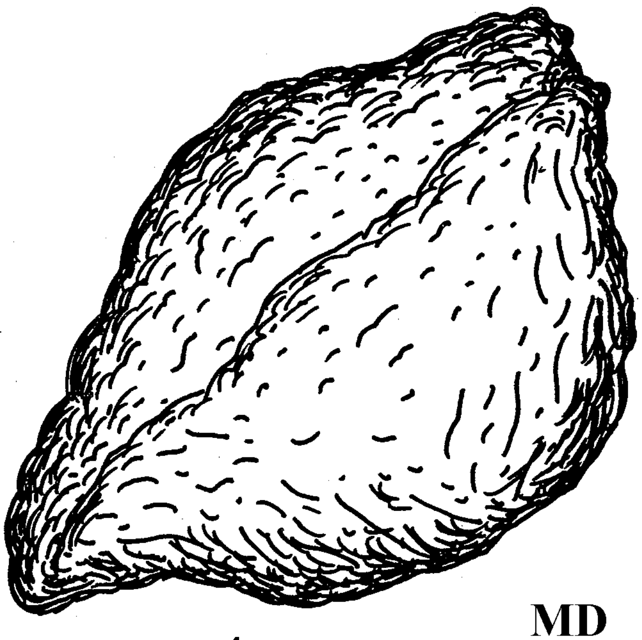

MD

4 Breast

Fish

1

2 OZ

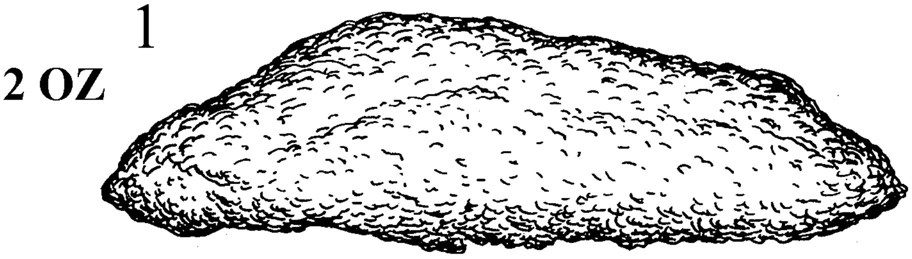

2

3 OZ

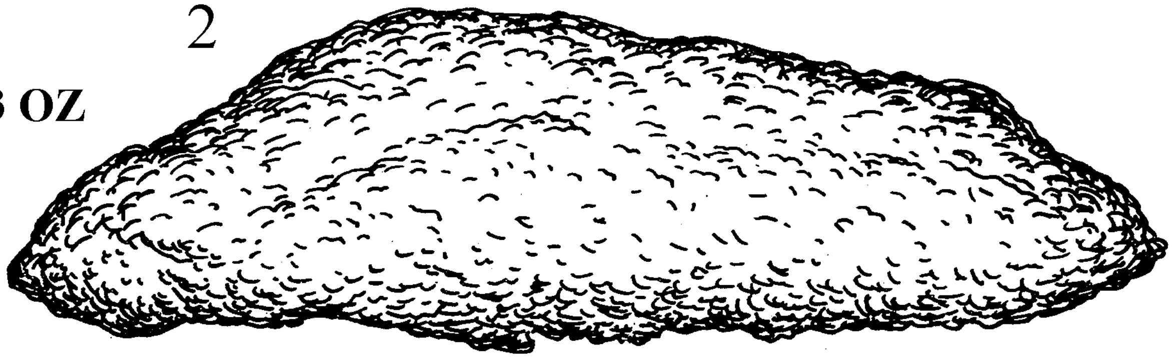

3

1/2 OZ

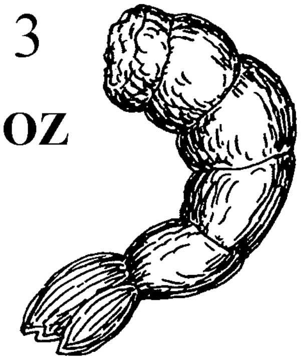

4

1 OZ

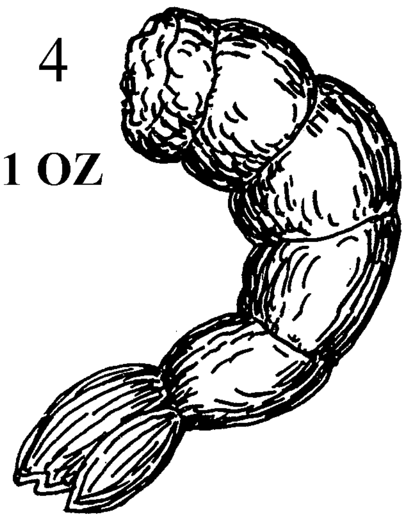

Adapted from: Van Horn LV, Stumbo P, Moag-Stahlberg A, Obarzanek E, Hartmuller VW, Farris RP, Kimm SY, Frederick M, Snetselaar L, Liu K. The Dietary Intervention Study in Children (DISC): dietary assessment methods for 8- to 10-year-olds. J Am Diet Assoc. 1993 Dec;93(12):1396-403.
Modified by Alejandra Valencia, Mary Stevens, Nutrition Coordinating Center, University of Minnesota for the Hispanic Community Health Study, Study of Latinos, 2007.
